# Hypoxia switches TET1 from being tumor-suppressive to oncogenic

**DOI:** 10.1038/s41388-023-02659-w

**Published:** 2023-04-05

**Authors:** Qi Yang, Hui Dang, Jiaxin Liu, Xingye Wang, Jingyuan Wang, Xinhui Lan, Meiju Ji, Mingzhao Xing, Peng Hou

**Affiliations:** 1 Key Laboratory for Tumor Precision Medicine of Shaanxi Province, The First Affiliated Hospital of Xi’an Jiaotong University, Xi’an, 710061 PR China; 2 Department of Endocrinology, The First Affiliated Hospital of Xi’an Jiaotong University, Xi’an, 710061 PR China; 3 Department of Otorhinolaryngology-Head and Neck Surgery, The First Affiliated Hospital of Xi’an Jiaotong University, Xi’an, 710061 PR China; 4 Department of Structural Heart Disease, The First Affiliated Hospital of Xi’an Jiaotong University, Xi’an, 710061 PR China; 5 Department of Clinical Laboratory, The First Affiliated Hospital of Xi’an Jiaotong University, Xi’an, 710061 PR China; 6 Center for Translational Medicine, The First Affiliated Hospital of Xi’an Jiaotong University, Xi’an, 710061 PR China; 7 School of Medicine, Southern University of Science and Technology, Shenzhen, 518055 Guangdong PR China

**Keywords:** Oncogenes, Cancer microenvironment

## Abstract

The classical oxidizing enzymatic activity of Ten Eleven Translocation 1 (TET1) and its tumor suppressor role are well known. Here, we find that high TET1 expression is associated with poor patient survival in solid cancers often having hypoxia, which is inconsistent with its tumor suppressor role. Through a series of in vitro and in vivo studies, using thyroid cancer as a model, we demonstrate that TET1 plays a tumor suppressor function in normoxia and, surprisingly, an oncogenic function in hypoxia. Mechanistically, TET1 mediates HIF1α-p300 interaction by acting as a co-activator of HIF1α to promote CK2B transcription under hypoxia, which is independent of its enzymatic activity; CK2 activates the AKT/GSK3β signaling pathway to promote oncogenesis. Activated AKT/GSK3β signaling in turn maintains HIF1α at elevated levels by preventing its K48-linked ubiquitination and degradation, creating a feedback loop to enhance the oncogenicity of TET1 in hypoxia. Thus, this study uncovers a novel oncogenic mechanism in which TET1 promotes oncogenesis and cancer progression through a non-enzymatic interaction between TET1 and HIF1α in hypoxia, providing novel therapeutic targeting implications for cancer.

## Introduction

Hypoxia in solid cancer is common, which can affect gene expression and molecular activities, generating a unique microenvironment that may profoundly affect cancer cellular functions and tumor behaviors [1, 2]. This may in turn influence the clinical outcomes of cancer and determine therapeutic targeting strategies for effective treatment. Some oxygen-sensitive enzymes, such as ten-eleven translocation (TET) enzymes, may be important players in such cellular microenvironment created by hypoxia in solid cancers. TET enzymes oxidize the 5-methyl group of cytosine to the stepwise derivatives, including 5-hydroxymethyl cytosine (5-hmC), and further oxidative products, representing the reversal process of DNA methylation [3–5]. Drawing particular attention is TET1, whose aberrant expression with corresponding 5-hmC levels has been reported in various cancers. As a DNA demethylator, TET1 has been known to play a broad tumor suppressor role through demethylating and activating tumor suppressor genes. TET1 has thus been extensively studied particularly for its function in the epigenetic reprogramming that plays a critical role in oncogenesis and tumor progression. Of great new interest and importance, some novel functions of TET1, beyond demethylation, have been recently unveiled, such as transcription factor recruitment, bivalent promoter induction and RNA modification, suggesting the existence of non-enzymatic biological activities of TET1 [6–9].

Loss of 5-hmC in cancer cells has been treated as a hallmark of cancer and used as a molecular biomarker for cancer diagnosis and poor prognosis [10–12]. There is abundant evidence showing that TET1 upregulates the expression of tumor suppressor genes by restoring 5-hmC levels of their promoter regions, thus playing tumor suppressor functions [13–15]. The tumor suppressor role of TET1 has been controversial, however [16–18]. For example, increased expression of TET1 has been actually linked to the chemoresistance of p53-deficient cancer cells [19] and impaired cellular responses to PI3K-mTOR inhibitors in triple-negative breast cancer [20]. Moreover, TET1 acts in human cancers in both 5-hmC-dependent and -independent manners and the distribution of 5-hmC is inconsistent with the change in TET1-induced expression profile [19, 21]. Thus, the functional spectrum of TET1 in human cancer remains to be fully defined.

Solid cancers commonly contain hypoxic microenvironments, with most tumors carrying a median oxygen level approaching 1% [22]. Compared with cell culture conditions routinely used for in vitro studies, the in vivo hypoxic environment may have important impacts on cancer cell behaviors and can be a reason for the discrepancy between preclinical findings and clinical manifestations of cancer [23]. Under hypoxic conditions, oxygen-dependent enzymes, such as hypoxia-inducible factor (HIF) prolyl hydroxylases (PHD), loose their catalytic activities. TETs are both 2-oxoglutarate (2-OG) and Fe^2+^-dependent dioxygenases, with a strict demand for molecular oxygen for their enzymatic activities. Although cell-free studies have found that TET1 is more tolerant of hypoxia than PHD [24], the enzyme activity of TET1 was reduced by 45% in human or mouse cells when cultured under 0.5% oxygen for 24 h [25]. Whether TET1 has non-enzymatic functions after the loss of its classical enzymatic activities in hypoxia is worth further exploring.

Here, we investigated possible oxygen state-dependent non-enzymatic functions of TET1 in cancer, which we found interestingly switch TET1 from being tumor-suppressive in normoxia to oncogenic in hypoxia. We took further step to identify the key molecules and define the molecular mechanism involved in this process, providing implications for novel therapeutic targeting potential in cancer.

## Results

### TET1 expression in different types of human cancers and its relationship with the patient survival

**Fig. 1:**
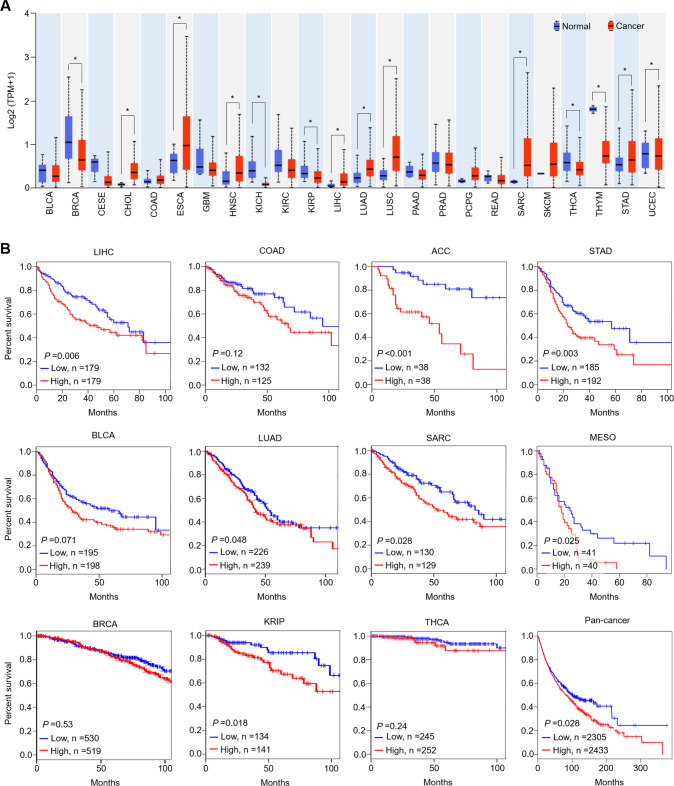
TET1 expression and its association with patient survival in different types of human cancers. **A** *TET1* mRNA expression in the indicated cancer tissues (red) and their corresponding normal tissues (control subjects, blue) (the data from TCGA dataset). Graph shows mean ± SD; unpaired Student’s test; *p < 0.05. **B** Kaplan–Meier curves showing the survival time in cancer patients with high and low *TET1* expression. Grouping was performed by median values of *TET1* TPM in the indicated cancer types using Log rank tests. TPM transcripts per million, BLCA bladder urothelial carcinoma, BRCA breast invasive carcinoma, CESC cervical squamous cell carcinoma and endocervical adenocarcinoma, CHOL cholangiocarcinoma, COAD colon adenocarcinoma, ESCA esophageal carcinoma, GBM glioblastoma multiforme, HNSC head and neck squamous cell carcinoma, KICH kidney chromophobe, KIRC kidney renal clear cell carcinoma, KIRP kidney renal papillary cell carcinoma, LIHC liver hepatocellular carcinoma, LUAD lung adenocarcinoma, LUSC lung squamous cell carcinoma, PAAD pancreatic adenocarcinoma, PRAD prostate adenocarcinoma, READ rectum adenocarcinoma, SARC sarcoma, SKCM skin cutaneous melanoma, THCA thyroid carcinoma, THYM thymoma, STAD stomach adenocarcinoma, UCEC uterine corpus endometrial carcinoma, ACC adrenocortical carcinoma, MESO mesothelioma.

We first analyzed *TET1* expression in different types of human cancers from The Cancer Genome Atlas (TCGA) database. The results showed that, compared with the control, *TET1* expression was significantly elevated in cholangiocarcinomas (CHOLs), esophageal carcinomas (ESCAs), head and neck squamous cell carcinomas (HNSCs), liver hepatocellular carcinomas (LIHCs), lung adenocarcinomas (LUADs), lung squamous cell carcinomas (LUSCs), sarcomas (SARCs) and stomach adenocarcinomas (STADs), while it was significantly downregulated in breast invasive carcinomas (BRCAs), kidney chromophobes (KICHs), kidney renal papillary cell carcinomas (KIRPs), thyroid carcinomas (THCAs) and thymomas (THYMs) (Fig. 1A). In our own samples, *TET1* expression was clearly downregulated in 17 primary thyroid cancers compared to their paired noncancerous tissues by qRT-PCR and immunohistochemistry (IHC) assays (Supplementary Fig. 1A, B). Moreover, on dot blot analysis of genomic 5-hmC in these samples, 5-hmC levels were significantly lower in cancer tissues than the control (Supplementary Fig. 1C), further supporting the above conclusion.

We next analyzed the association of *TET1* expression with patient survival using the TCGA dataset and found, surprisingly, that high *TET1* expression was strongly associated with poor patient survival, even in some cancers where its expression was significantly downregulated compared to the control (Fig. 1B). We also performed a pan-cancer analysis, which validated this conclusion (Fig. 1B).

### TET1 plays distinct roles in thyroid cancer in vitro and in vivo

**Fig. 2:**
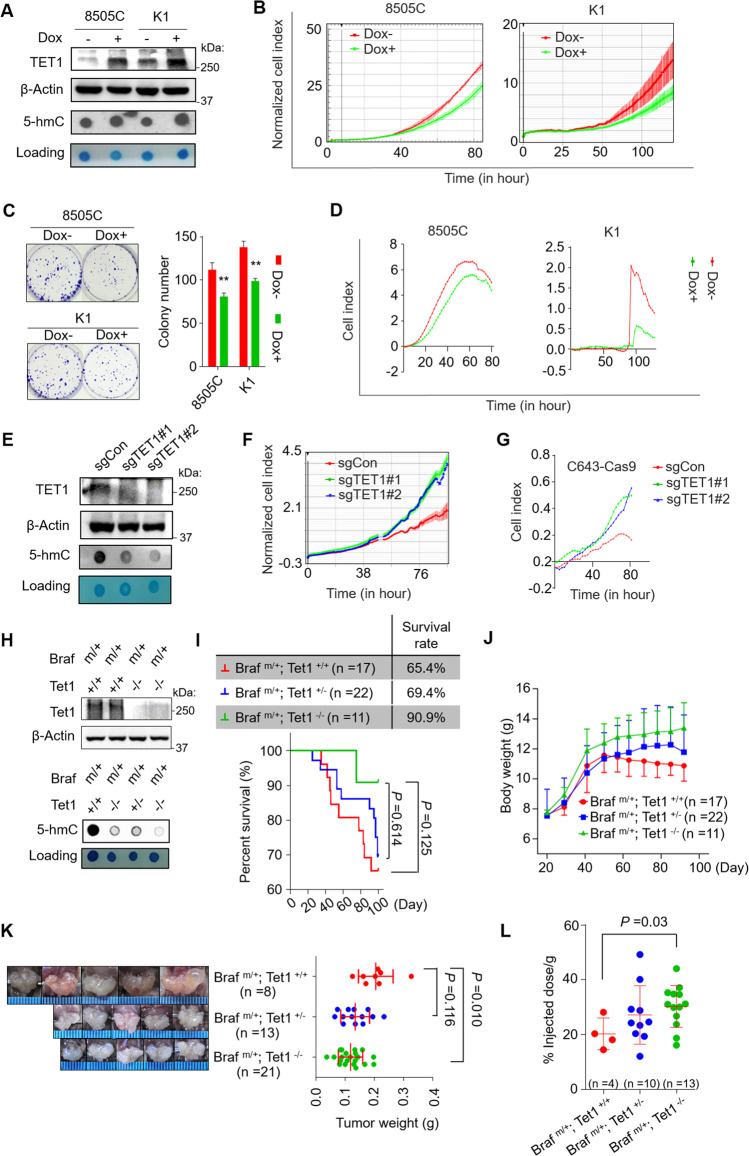
Distinct biological roles of TET1 in thyroid cancer in vitro and in vivo. **A** Western blot analysis of TET1 and dot blot of genome 5-hmC in 8505C and K1 cells treated with/without Dox. Methylene blue staining of the membrane showing the DNA loading. Cell proliferation (**B**), colony formation (**C**) and cell migration (**D**) assays were performed to examine the effect of ectopic expression of TET1 in 8505C and K1 cells by doxycycline (Dox)-inducible gene expression system (Tet-On). **E** Western blot and dot blot assays were performed for TET1-knockout C643 cells and control cells. A real-time cell analyzer (RTCA) was used to monitor the proliferation (**F**) and migration (**G**) of the above cells. **H** Western blot and dot blot assays showing Tet1 expression and 5-hmC levels in mice with different genotypes. **I** Survival rates (table in the upper panel) and survival curves (lower panel) of mice with different genotypes during the 100-day breeding. **J** Body weight of the indicated mice. Plots and error bars indicate the mean and SD values recorded weekly. **K** Representative pictures and weight of thyroid tumors at the 100th day. **L** Comparison of iodine uptake rate of thyroid in mice with different genotypes. Dox− negative control, Dox+ cells inducibly expressing TET1. sgCon control cells, sgTET1 TET1-knockout C643 cells. Braf^m/+^ *Braf* heterozygous mutation, Tet1^+/+^ *Tet1* wild-type, Tet1^*−/−*^ *Tet1* homozygous deletion. Graph shows mean ± SD. Statistical analysis was performed by unpaired Student’s test. **p < 0.01.

The above results seemed contradictory. To clarify it, we took thyroid cancer as an example model to perform a series of in vitro and in vivo experiments. We ectopically expressed TET1 in thyroid cancer cell lines 8505C and K1 using the doxycycline (dox)‐inducible Tet‐On system and demonstrated that ectopic expression of TET1 upregulated genomic 5-hmC levels compared with the control (Fig. 2A). Ectopic expression of TET1 significantly inhibited the proliferation in 8505C and K1 cells compared with the control (Fig. 2B). This was further supported by colony formation and cell migration assays (Fig. 2C, D). We knocked out TET1 in thyroid cancer cell line C643 using the lentivirus-mediated CRISPR-Cas9 system and successfully validated TET1 knockout in single-cell colonies by Sanger sequencing, western blot analysis and dot-blot analysis of genomic 5-hmC (Supplementary Table 1 and Fig. 2E). This TET1 knockout dramatically promoted cell proliferation and migration compared with the control (Fig. 2F, G). These in vitro results indicated that TET1 functioned as a tumor suppressor in thyroid cancer cells, which was consistent with a recent study suggesting that TET1 inhibited thyroid cancer cell migration and invasion [26].

To study the in vivo role of TET1 in thyroid cancer, we generated thyroid-specific *Tet1* knockout mouse model by crossing *LSL-Braf*^*V600E*^, *LSL-Tet1* and thyroid peroxidase (*Tpo*)-*Cre* mice to generate thyroid cancer with various genotypes (Supplementary Fig. 2A). We validated the successful knockout of *Tet1* in thyroid glands using western blot analysis and dot-blot analysis of genomic 5-hmC and IHC assay (Fig. 2H and Supplementary Fig. 2B). We found that the best survival was seen in mice with homozygous knockout of *Tet1* (*Braf*^*m/+*^*; Tet1*^*−/−*^), second in mice with heterozygous knockout of *Tet1* (*Braf*^*m/+*^*; Tet1*^*+/−*^), and least in mice with wild-type *Tet1* (*Braf*^*m/+*^*; Tet1*^*+/+*^) (Fig. 2I). Moreover, *Braf*^*m/+*^*/Tet1*^*−/−*^ mice were more capable of maintaining body weight gain compared with *Braf*^*m/+*^*; Tet1*^*+/−*^ and *Braf*^*m/+*^*; Tet1*^*+/+*^ mice (Fig. 2J). The data also showed that tumor volume and weight in *Braf*^*m/+*^*; Tet1*^*−/−*^ mice were significantly smaller than those of *Braf*^*m/+*^*; Tet1*^*+/+*^ mice (Fig. 2K). These results were also supported by Ki-67 staining (Supplementary Fig. 2C).

Given that *Braf*^*V600E*^ can drive dedifferentiation and poor iodine uptake of thyroid follicular epithelial cells [27, 28], we next tested the effect of *Tet1* knockout on the uptake of radioiodine in these transgenic mice and found that *Braf*^*m/+*^*; Tet1*^*−/−*^ mice exhibited stronger ability to take up radioiodine than *Braf*^*m/+*^*; Tet1*^*+/−*^ and *Braf*^*m/+*^*; Tet1*^*+/+*^ mice, especially the latter (Fig. 2L), suggesting that the thyroid tumor was more differentiated (less aggressive) in *Braf*^*m/+*^*; Tet1*^*−/−*^ mice. Correspondingly, *Braf*^*m/+*^*; Tet1*^*−/−*^ mice also exhibited the higher expression of sodium iodine transporter (NIS) compared with *Braf*^*m/+*^*; Tet1*^*+/−*^ and *Braf*^*m/+*^*; Tet1*^*+/+*^ mice (Supplementary Fig. 2D), further supporting the above conclusion. Collectively, these in vivo findings support a tumor-promoting role of Tet1 in thyroid cancer and suggest that TET1 plays distinct roles in thyroid cancer in vivo versus in vitro.

### Hypoxia switches on the oncogenic mode of TET1 via HIF1α

**Fig. 3:**
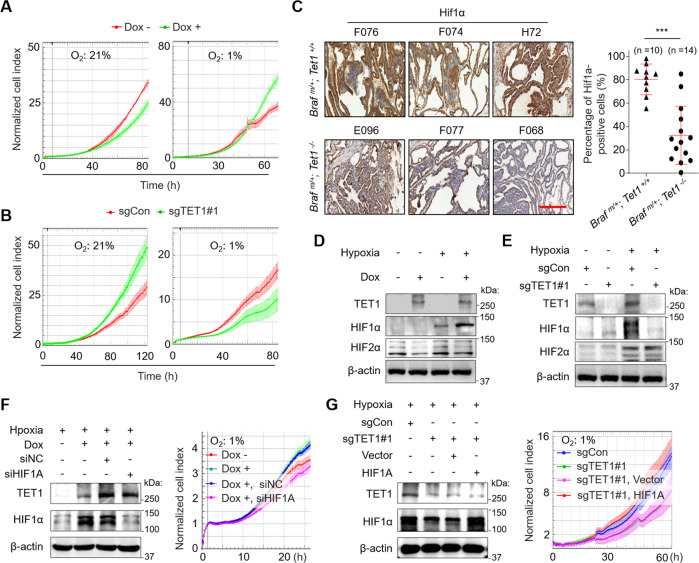
HIF1α is required for the promoting effect of TET1 on thyroid cancer cell proliferation under hypoxia. **A** The proliferation of 8505C cells inducibly expressing TET1 and control cells under normoxia and hypoxia. **B** The proliferation of TET1-knockout C643 cells and control cells under both normoxia and hypoxia. **C** The IHC staining of HIF1α and its quantification in thyroid cancer tissues of the indicated mice. **D** Western blot analysis of TET1, HIF1α and HIF2α in 8505C cells inducibly expressing TET1 and control cells under normoxia and hypoxia. **E** Western blot analysis of TET1, HIF1α and HIF2α in TET1-knockout C643 cells and control cells under both normoxia and hypoxia. Western blot (left panel) and proliferation (right panel) of 8505C (**F**) and C643 (**G**) cells with the indicated treatments. Dox− negative control, Dox+ cells inducibly expressing TET1. siNC cells transfected with control siRNA, siHIF1A cells transfected with siRNA against HIF1α. sgCon control cells, sgTET1 TET1-knockout C643 cells. Vector, cells transfected with empty plasmid, HIF1A cells transfected with the plasmid expressing HIF1α. Scale bars in IHC pictures, 50 μm. Graph shows mean ± SD; unpaired Student’s test; **p < 0.01. Braf^m/+^ *Braf* heterozygous mutation, Tet1^+/+^ *Tet1* wild-type, Tet1^*−/−*^ *Tet1* homozygous deletion.

We next explored the mechanism underlying the above phenomenon. We hypothesized that hypoxia might govern the discrepant results between in vitro and in vivo studies. To test this, we cultured thyroid cancer cells in both normoxia (21% O_2_) and hypoxia (1% O_2_) and found that ectopic expression of TET1 in 8505C cells did suppress cell proliferation under normoxia, while it promoted cell proliferation under hypoxia (Fig. 3A). Conversely, knocking out TET1 in C643 cells promoted cell proliferation under normoxia, while it suppressed cell proliferation under hypoxia (Fig. 3B). These results again suggested that TET1 displayed a tumor-suppressive function in normoxia and an oncogenic function in hypoxia.

Given that HIF1α and HIF2α are master regulators of cellular response to hypoxia [29], we first examined their expression in thyroid tissues of normal mice and tumor tissues of *Braf*^*V600E*^ transgenic mice by the IHC assay. The results showed that HIF1α and HIF2α staining was positive in the latter, while they were negative in the former (Supplementary Fig. 3A). Interestingly, we found that HIF1α expression, rather than HIF2α expression, was significantly decreased in tumor tissues of *Braf*^*m/+*^*; Tet1*^*−/−*^ mice compared with that of *Braf*^*m/+*^*; Tet1*^*+/+*^ mice (Fig. 3C and Supplementary Fig. 3B). We next tested the effect of ectopic expression or deletion of TET1 on HIF1α expression in thyroid cancer cells. As shown in Fig. 3D, E, we were unable to detect HIF1α in 8505C and C643 cells under normoxia regardless of TET1 status. However, under hypoxia, ectopic expression of TET1 upregulated HIF1α expression, while TET1 knockout downregulated HIF1α expression. Unlike HIF1α, ectopic expression or knockout of TET1 almost did not affect protein expression of HIF2α in these two cell lines (Fig. 3D, E). Besides, the activity of HIF1α responsive element (HRE) was significantly enhanced by TET1 overexpression (Supplementary Fig. 3C), while it was decreased by TET1 knockout, both only under hypoxia (Supplementary Fig. 3D). Also, we found that hypoxia dramatically induced the expression of the HIF1α downstream targets *HK2* and *GLUT1*, while overexpression or knockout of TET1 correspondingly upregulated or downregulated their expression (Supplementary Fig. 3E, F).

As stated above, we speculated that HIF1α might mediate oncogenic roles of TET1 in thyroid cancer. Indeed, we found that HIF1α knockdown clearly attenuated the promoting effect of TET1 overexpression on cell proliferation compared with the control in 8505C cells under hypoxia (Fig. 3F). Conversely, HIF1α overexpression effectively mitigated the inhibitory effect of TET1 knockout on cell proliferation (Fig. 3G). These results suggest that TET1 upregulates HIF1α expression and hypoxia switches on its oncogenic modes via HIF1α.

### TET1 stabilizes HIF1α by activating the AKT/GSK3β signaling pathway

**Fig. 4:**
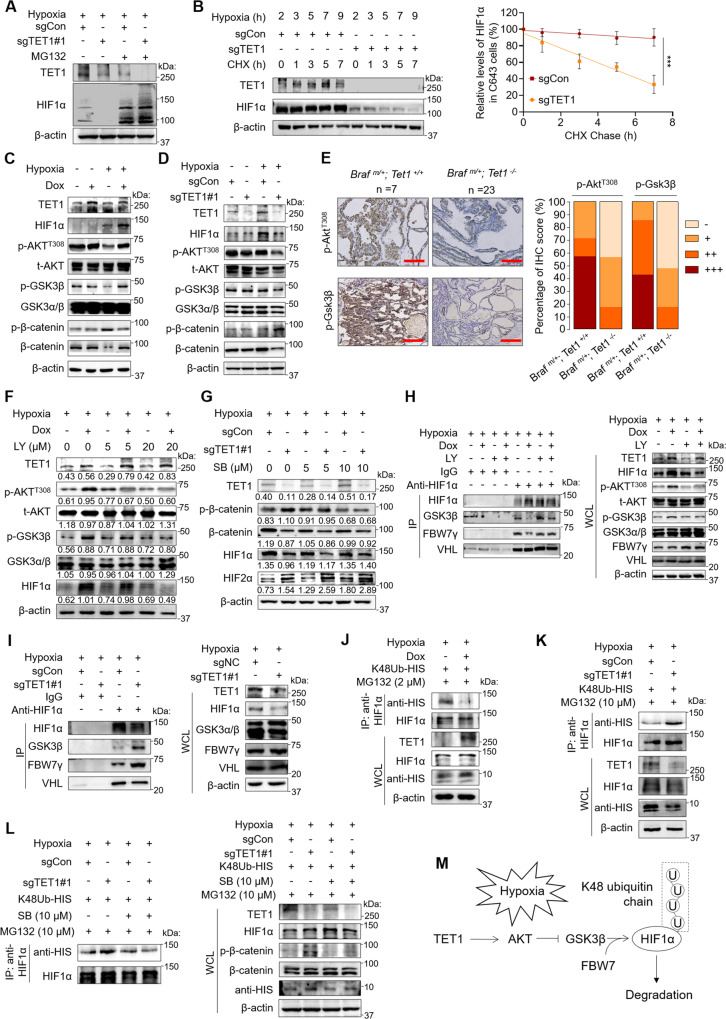
HIF1α is stabilized by TET1 via the activation of AKT/GSK3β pathway. **A** Western blot analysis of TET1 and HIF1α in C643 cells with the indicated treatments under hypoxia. MG132 concentration: 10 μM. **B** Western blot analysis of TET1 and HIF1α in 8505C cells with the indicated treatments. The band intensity of HIF1α was normalized to that of β-actin, and subsequently normalized to that of cells treated without CHX. Data are shown as the mean ± SD. CHX cycloheximide at 200 μg/ml. Western blot analysis of TET1, HIF1α, p-AKT^T308^, t-AKT, p-GSK3β, GSK3α/β, p-β-catenin and β-catenin in 8505C (**C**) and C643 (**D**) cells with the indicated treatments. **E** The IHC staining for p-Akt and p-Gsk3β (left panel) and their quantification (right panel) in mice with different genotypes. **F** Western blot analysis of TET1, p-AKT^T308^, t-AKT, p-GSK3β, GSK3α/β and HIF1α in 8505C cells with the indicated treatments. LY PI3K inhibitor LY294002 at the indicated concentration. The densitometry ratio of the indicated proteins to β-actin (loading control) was shown below the corresponding band. **G** Western blot analysis of TET1, p-GSK3β^S9^, GSK3α/β, HIF1α and HIF2α in C643 cells with the indicated treatments. SB GSK3 inhibitor SB-216763 at the indicated concentration. The densitometry ratio of the indicated proteins to β-actin (loading control) was shown below the corresponding band. **H** Co-immunoprecipitation (Co-IP) assay showing the interaction of HIF1α with GSK3β, FBW7γ and VHL in 8505C cells with the indicated treatments. LY cells treated with 20 μM LY294002 for 24 h. **I** Co-IP assay showing the interaction of HIF1α with GSK3β, FBW7γ and VHL in C643 cells with the indicated treatments. In vitro protein ubiquitination analysis of the K48-linked ubiquitination of HIF1α in 8505C (**J**) and C643 (**K**) cells with the indicated treatments. Anti-His tag was used to show the K48-only His-tagged ubiquitin. **L** K48-linked ubiquitination of HIF1α in C643 cells with the indicated treatments. The IP shows K48-only His-tagged ubiquitin and HIF1α in the precipitants (left panel). Cells were treated with SB and MG132 at the indicated concentration for 24 h. **M** A schematic model summarizing the mechanism of TET1 stabilizing HIF1α via the activation the AKT/GSK3β pathway under hypoxia. Braf^m/+^ *Braf* heterozygous mutation, Tet1^+/+^ *Tet1* wild-type, Tet1^*−/−*^ *Tet1* homozygous deletion. Scale bare in IHC pictures, 50 μm. Sections were rank scored [“negative” (−), “weak” (+), “moderate” (++) and “strong” (+++)]. Graph shows the proportion of each score for the indicated groups. Dox− negative control, Dox+ cells inducibly expressing TET1, sgCon control cells, sgTET1 TET1-knockout C643 cells, p-AKT^T308^ phosphorylated AKT at T308, p-GSK3β phosphorylated GSK3β at S9, p-β-catenin phosphorylated β-catenin at S33/37/T41, WCL whole cell lysates.

We next investigated how TET1 upregulated HIF1α by examining the transcription of *HIF1A* upon overexpression or knockout of TET1. The results showed that change in TET1 did not affect the transcription of *HIF1A* (Supplementary Fig. 4A, B). However, the proteasome inhibitor MG132 was able to remarkably reverse the inhibitory effect of TET1 knockout on HIF1α protein expression under hypoxia (Fig. 4A). Moreover, using cycloheximide (CHX) to block new protein synthesis under hypoxia, we found that knockout of TET1 in C643 cells impaired HIF1α protein stability compared with the control (Fig. 4B). Conversely, ectopic expression of TET1 in 8505C cells inhibited the turnover of HIF1α proteins compared with the control (Supplementary Fig. 4C). These findings suggested that TET1 enhanced the protein stability of HIF1α rather than altering its transcription under hypoxia; such regulation of the HIF1α protein stability by TET1 occurred in an oxygen-independent manner. There has been a study suggesting that GSK3β phosphorylates and triggers E3 ubiquitin ligase FBW7-mediated ubiquitination and degradation of HIF1α in an oxygen-independent manner [30]. Thus, we examined the effect of TET1 on the activity of AKT/GSK3β pathway. As shown in Fig. 4C, TET1 overexpression in 8505C cells increased the phosphorylation of AKT at T308 under hypoxia, while this effect was not seen under normoxia. Moreover, we found that, as a key downstream event of PI3K/AKT pathway signaling, the phosphorylation of GSK3β at S9 was increased upon TET1 overexpression under hypoxia but not normoxia, indicating that TET1 inhibited the GSK3β activity only under hypoxia. This was further suggested by decreased phosphorylation (S33/37/T41) and increased expression of β-catenin. Conversely, TET1 knockdown in C643 cells showed an inhibitory effect on the AKT/GSK3β/β-catenin pathway under hypoxia, but not under normoxia (Fig. 4D). This was also supported by IHC staining for the phosphorylation of Akt at T308 and Gsk3β at S9 in tumor tissues of *Braf*^*m/+*^*; Tet1*^*−/−*^ and *Braf*^*m/+*^*; Tet1*^*+/+*^ mice (Fig. 4E). These results suggest that hypoxia switches on the function of TET1 to activate the AKT/GSK3β signaling pathway.

The activity of AKT/GSK3β pathway and HIF1α protein expression promoted by TET1 overexpression in 8505C cells under hypoxia could be attenuated by treatment with the PI3K inhibitor LY294002 (Fig. 4F). On the other hand, treatment with the GSK3β inhibitor SB-216763 could partially reverse the inhibitory effect of TET1 knockout on protein expression of β-catenin and HIF1α in C643 cells under hypoxia (Fig. 4G). Moreover, among the three *FBW7* gene products, FBW7γ was demonstrated to interact with HIF1α in both 8505C and C643 cells under hypoxia (Supplementary Fig. 4D, E). TET1 overexpression impaired the interaction between HIF1α and GSK3β or FBW7γ under hypoxia, while this effect was reversed by LY294002 treatment (Fig. 4H). Conversely, TET1 knockout enhanced the interaction of HIF1α with GSK3β or FBW7γ under hypoxia (Fig. 4I). In contrast, overexpression or knockout of TET1 virtually had no effect on the interaction between HIF1α and VHL (Fig. 4H, I), further supporting that TET1 regulated HIF1α protein stability in an oxygen-independent manner.

The above findings encouraged us to examine the role of TET1 in the ubiquitination of HIF1α proteins. The results showed that TET1 overexpression increased the polyubiquitination of HIF1α (Supplementary Fig. 4F), which was consistent with a previous study [31]. Considering that multifaceted ubiquitin chains have been recognized to encode different functions, such as protein degradation, protein trafficking, and DNA repairment [32] and K48-linked polyubiquitination is a major characteristic of FBW7 substrates [33–35], we performed in vitro protein ubiquitination assays with a His-tagged K48-only ubiquitin plasmid. The results showed that TET1 overexpression in 8505C cells robustly decreased K48-linked polyubiquitination of HIF1α under hypoxia (Fig. 4J). Conversely, TET1 knockout in C643 cells increased K48-linked polyubiquitination compared with the control (Fig. 4K) and this effect could be reversed by the GSK3β inhibitor SB-216763 (Fig. 4L). These results, taken together, demonstrate that TET1 stabilizes HIF1α by suppressing its FBW7γ-mediated K48-linked ubiquitination and proteasome degradation via the AKT/GSK3β pathway in thyroid cancer cells (Fig. 4M).

### TET1 activates the PI3K/AKT pathway by upregulating Casein Kinase 2 (CK2)

**Fig. 5:**
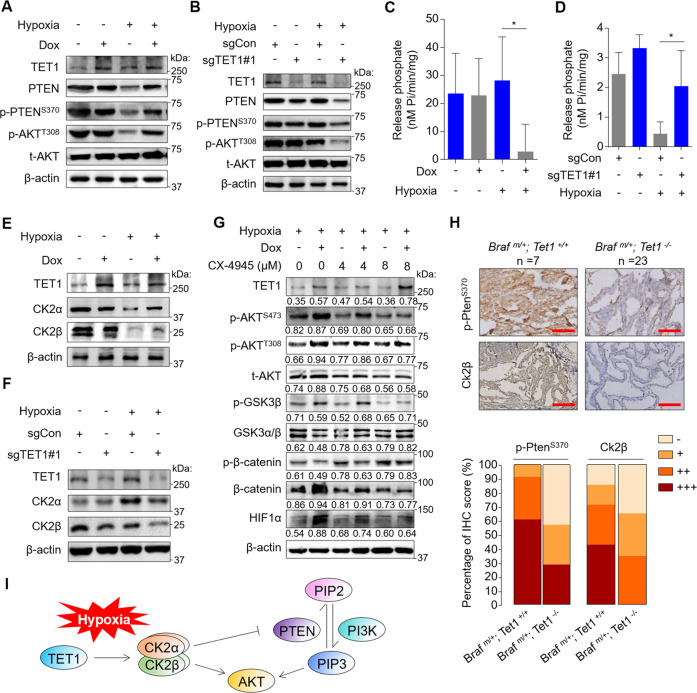
TET1 activates AKT signaling by the CK2-mediated PTEN inactivation. Western blot analysis of TET1, p-PTEN^S370^, PTEN, p-AKT^T308^ and t-AKT in 8505C (**A**) and C643 (**B**) cells with the indicated treatments. The PIP3 phosphatase assay was performed to detect PTEN activity in 8505C (**C**) and C643 (**D**) cells with the indicated treatments (*n* = 3 biological replicates). The idle PTEN activity was shown as mean ± SD; unpaired Student’s test; *p < 0.05. Western blot analysis of CK2α and β in 8505C (**E**) and C643 (**F**) cells with the indicated treatments. **G** Western blot analysis of TET1, p-AKT^S473^, p-AKT^T308^, t-AKT, p-GSK3β, GSK3α/β, p-β-catenin, β-catenin and HIF1α in 8505C cells with the indicated treatments. Cells were treated with CK2 inhibitor CX-4945 at the indicated concentration for 24 h. The densitometry ratio of the indicated proteins to β-actin (loading control) was shown below the corresponding band. **H** The IHC staining for p-Pten at S370 and Ck2β (upper panel) and their quantification (lower panel) in mice with the indicated genotypes. **I** A schematic model concluding that TET1 activates AKT signaling by the CK2-mediated PTEN inactivation under hypoxia. Dox− negative control, Dox+ cells inducibly expressing TET1, sgCon control cells, sgTET1 TET1-knockout C643 cells. Scale bare in IHC pictures, 50 μm. Braf^m/+^ *Braf* heterozygous mutation, Tet1^+/+^ *Tet1* wild-type, Tet1^*−/−*^ *Tet1* homozygous deletion. Rank scores were estimated [i.e., “negative” (−), “weak” (+), “moderate” (++), and “strong” (+++)]. Graph shows the proportion of each score for different groups.

To explore the mechanism that TET1 activated AKT under hypoxia, we examined the effect of TET1 on the expression and activity of PTEN, a negative regulator of PI3K/AKT pathway [36]. The results showed that TET1 overexpression in 8505C cells increased PTEN expression and the phosphorylation of PTEN at S370 and AKT at T308 under hypoxia, but not under normoxia (Fig. 5A). The opposite was observed with TET1 knockout in C643 cells, further supporting this conclusion (Fig. 5B). This seemed to be a contradiction between increased expression of PTEN and AKT activation. However, Casein Kinase 2 (CK2)-mediated phosphorylation of C-terminal residues (such as S370) of PTEN has been demonstrated to protect PTEN from degradation but fail to maintain its lipid phosphatase activity [37, 38]. This was supported by the above results (Fig. 5A, B). Furthermore, CK2 can also directly phosphorylate AKT at S129, resulting in its full activation [39].

Given the above findings, we performed phosphatidylinositol-3,4,5-trisphosphate (PIP3) phosphatase assays to validate the effect of TET1 on PTEN activity in thyroid cancer cells. The results showed that TET1 overexpression in 8505C cells strongly suppressed PTEN activity (Fig. 5C), while TET1 knockout in C643 cells increased PTEN activity under hypoxia but not under normoxia (Fig. 5D). Considering that CK2 mediates the phosphorylation and inactivation of PTEN [37, 38], we next tested the effect of TET1 on the expression of α and β subunits of CK2 (CK2α and CK2β). The results showed that TET1 overexpression upregulated the expression of CK2α and CK2β (Fig. 5E), while TET1 knockout downregulated their expression under hypoxia but not under normoxia (Fig. 5F). We treated 8505C cells with the CK2 inhibitor CX-4945 and found robust suppression of the AKT/GSK3β pathway and loss of the stabilizing effect of TET1 on HIF1α (Fig. 5G). Besides, we performed IHC assays and validated the above conclusions in a transgenic mouse model by staining phosphorylated Pten (S370), Ck2α and Ck2β (Fig. 5H and Supplementary Fig. 5). Collectively, we conclude that TET1 stabilizes HIF1α under hypoxia by activating the CK2/AKT/GSK3β signaling axis (Fig. 5I).

### TET1 enhances the CK2B transcription as a coactivator of HIF1α

**Fig. 6:**
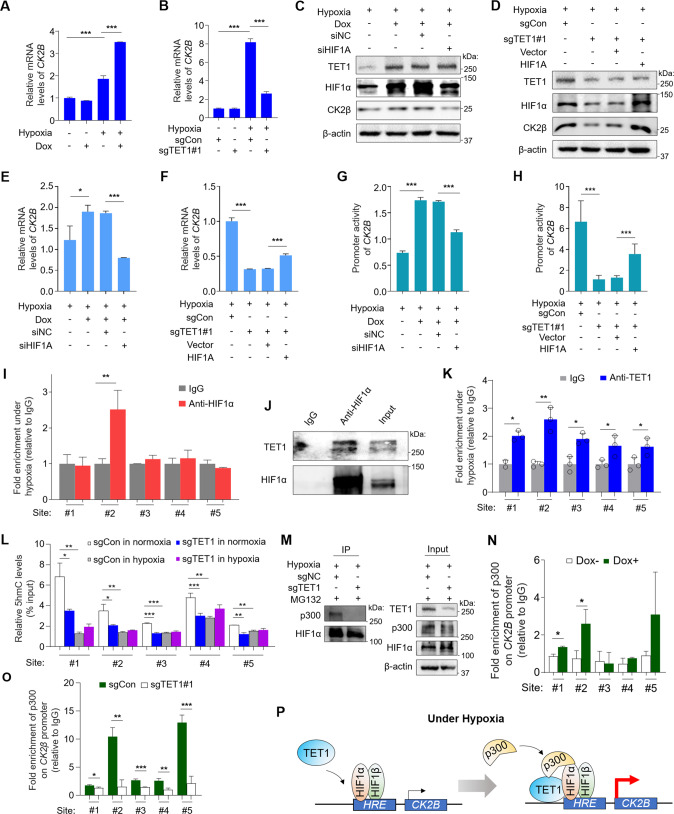
TET1 acts as a coactivator of HIF1α to promote *CK2B* transcription. *CK2B* mRNA expression in 8505C (**A**) and C643 (**B**) cells with the indicated treatments by qRT-PCR assay (*n* = 3 biological replicates). Western blot analysis of TET1, HIF1α and CK2β in 8505C (**C**) and C643 (**D**) cells with the indicated treatments. *CK2B* mRNA expression in 8505C (**E**) and C643 (**F**) cells with the indicated treatments by qRT-PCR assay (*n* = 3 biological replicates). Dual-luciferase reporter system was performed to determine *CK2B* promoter activity in 8505C (**G**) and C643 (**H**) cells with the indicated treatments (*n* = 3 biological replicates). **I** ChIP-qPCR assay showing the binding of HIF1α to *CK2B* promoter in 8505C cells under hypoxia (*n* = 3 biological replicates). **J** Co-IP assay showing the interaction between HIF1α and TET1 in C643 cells under hypoxia. **K** ChIP-qPCR assay showing the binding of TET1 to *CK2B* promoter in C643 cells under hypoxia (*n* = 3 biological replicates). **L** hMeDIP-qPCR assay of *CK2B* promoter in C643 cells with the indicated treatments (*n* = 3 biological replicates). **M** Co-IP assay showing interaction between HIF1α and p300 in C643 cells with the indicated treatments. ChIP-qPCR assay showing the binding of p300 to *CK2B* promoter in 8505C (**N**) and C643 (**O**) cells under hypoxia (*n* = 3 biological replicates). **P** A schematic model describing that TET1 promotes *CK2B* transcription as a coactivator of HIF1α by mediating p300-HIF1α interaction under hypoxia. For mRNA analysis, *GAPDH* was used as a reference gene. Dox− negative control, Dox+ cells inducibly expressing TET1, sgCon control cells, sgTET1 TET1-knockout C643 cells. siNC cells transfected with control siRNA, siHIF1A cells transfected with siRNA against HIF1α, Vector cells transfected with empty plasmid, HIF1A cells transfected with the plasmid expressing HIF1α. For ChIP-qPCR assays, graph shows enrichment folds of anti-HIF1α, anti-TET1, and anti-p300 relative to IgG. For hMeDIP-qPCR, graph shows the percentage of hydroxylmethylated DNA in genomic DNA. Histograms were shown in mean ± SD. Statistical analysis was performed by unpaired Student’s test. *p < 0.05, **p < 0.01, and ***p < 0.001.

We next investigated the mechanism that TET1 upregulated CK2α and β. It is well known that two molecules of each CK2α and β form the tetrameric holoenzyme of CK2. The former is catalytically active, while the latter is responsible for the stabilization and localization of the kinase [40, 41]. Although the above results demonstrated that the protein expression of CK2α was elevated by TET1 under hypoxia, this did not occur at the transcriptional level (Supplementary Fig. 6A, B). In contrast, hypoxia significantly increased mRNA expression of *CK2B* and TET1 overexpression further increased its expression, while TET1 knockout dramatically decreased its expression under hypoxia (Fig. 6A, B). Also, knockdown of HIF1α in 8505C cells attenuated the promoting effect of TET1 overexpression on CK2β expression compared with control cells (Fig. 6C). Conversely, ectopic expression of HIF1α in TET1-knockout C643 cells completely reversed the inhibitory effect of TET1 knockout on CK2β expression (Fig. 6D), as also supported by the results of qRT-PCR assays (Fig. 6E, F). These findings suggest that TET1 promotes the *CK2B* transcription under hypoxia in a HIF1α-dependent manner.

To further support the above conclusion, we performed luciferase reporter assays and, as expected, found that TET1 overexpression significantly increased the promoter activity of *CK2B* under hypoxia, while HIF1α knockdown showed an opposite effect (Fig. 6G). Conversely, TET1 knockout decreased the promoter activity of *CK2B* under hypoxia, while HIF1α overexpression partially reversed this effect (Fig. 6H). Thus, we speculated that *CK2B* might be a potential downstream target of HIF1α. To prove this, we performed chromatin immunoprecipitation (ChIP) assays in 8505C cells under hypoxia using anti-HIF1α antibody, followed by quantitative PCR (qPCR) targeting multiple HRE consensus sequences (RCGTG) within the *CK2B* promoter. One of five fragments of the *CK2B* promoter was significantly enriched in this ChIP assay (Fig. 6I), demonstrating that *CK2B* is a direct downstream target of HIF1α.

TET1 has been demonstrated to be a coactivator of HIF2α, depending on its demethylation activity [17]. To determine whether there was a similar relationship between TET1 and HIF1α, we performed co-IP assays in C643 cells under hypoxia and demonstrated the interaction between them (Fig. 6J). We also showed that TET1 was enriched to HREs within the *CK2B* promoter by ChIP-qPCR and ChIP-PCR assays in C643 cells under hypoxia (Fig. 6K and Supplementary Fig. 6C), further supporting the above conclusion. To determine whether the demethylation activity of TET1 was essential for *CK2B* transcription under hypoxia, we preformed hydroxymethyl DNA immunoprecipitation (hMEDIP) assays to test the effect of hypoxia or TET1 knockout on 5-hmC distribution within the *CK2B* promoter. As shown in Fig. 6L, TET1 knockout under normoxia significantly reduced 5-hmC levels across the entire promoter relative to the control, while hypoxia eliminated this effect and, expectedly, reduced 5-hmC levels. These data suggest that TET1 acts as a coactivator of HIF1α to promote *CK2B* transcription in an enzyme-independent manner.

Considering non-catalytic functions of TET1, such as protein recruitment, we proposed that TET1 might participate in the HIF1α-driven and transcriptionally active complex consisting of CBP/p300. To examine a role of TET1 in the stability of this complex, we performed co-IP assays in MG132-treated C643 cells under hypoxia and found that TET1 knockout disrupted the interaction between HIF1α and p300 compared with the control (Fig. 6M). Moreover, ChIP-qPCR assays under hypoxia showed that TET1 overexpression in 8505C enhanced the binding of p300 to the *CK2B* promoter (Fig. 6N), while TET1 knockout in C643 cells strongly diminished the enrichment of p300 on multiple HREs within the *CK2B* promoter (Fig. 6O). These results indicate that TET1 integrates and stabilizes the transcriptionally active complex of HIF1α recognizing HREs within the *CK2B* promoter, thereby acting as a coactivator of HIF1α to promote *CK2B* transcription (Fig. 6P).

**Fig. 7:**
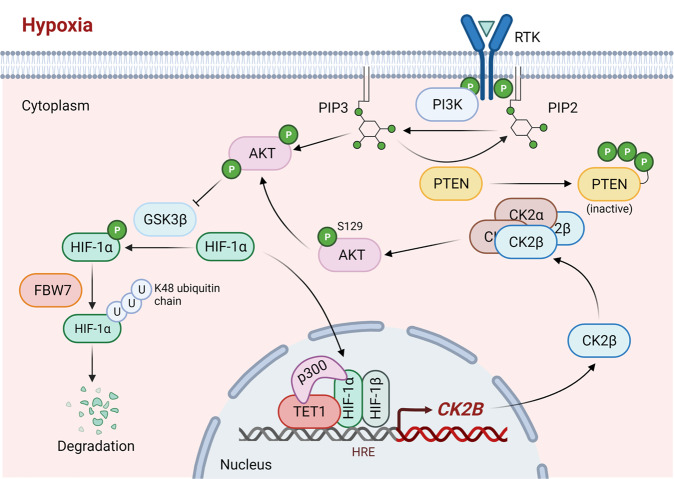
A schematic model summarizing the oncogenic roles of TET1 in thyroid cancer under hypoxia. Briefly, under hypoxia, TET1 promotes *CK2B* expression by stabilizing the transcriptional active complex of HIF1α/p300. In turn, CK2β facilitates the integration and enzyme maximization of CK2 to fully activate AKT signaling via phosphorylation and inactivation of PTEN. Activated AKT cascade prevents the FBW7-mediated K48-linked ubiquitination and degradation of HIF1α, thereby forming a positive CK2/AKT/GSK3β/HIF1α feedback loop to accelerate tumor progression.

Based on the above results, we proposed a model to illustrate how TET1 plays an oncogenic role in thyroid cancer cells under hypoxia (Fig. 7). Briefly, under hypoxia, TET1 promotes *CK2B* transcription by stabilizing HIF1α/p300 complex docking on its promoter. CK2β at increased expression phosphorylates and inactivates PTEN by forming a heterotetramer with CK2α, thereby activating the PI3K/AKT/GSK3β pathway, which prevents FBW7γ-mediated K48-linked ubiquitination and degradation of HIF1α. This forms a feedback loop in which TET1 promotes thyroid cancer progression via the CK2/AKT/GSK3β/HIF1α signaling pathway.

Given the core role of CK2 in the above feedback loop, we next tested the therapeutic potential of targeting CK2 using CK2 inhibitors to block the tumor-promoting effect of TET1 under hypoxia in cancer. Indeed, as shown in Supplementary Fig. 6D, E, CK2 inhibitor CX-4945 significantly attenuated the pro-proliferative and pro-migratory effects of ectopic expression of TET1 in 8505C cells under hypoxia. These results, taken together, suggest that CK2 inhibitors have the potential to effectively treat solid cancer, including thyroid cancer, with high TET1 expression.

## Discussion

Dynamic changes of oxygenation represent a major component of tumor microenvironment that reprogram cancer cells for adaptive survival, clone selection and development [42, 43]. Hypoxia is a hallmark of many solid tumors, being closely associated with poor patient prognosis, therapy resistance, and invasion/metastasis [44]. As a sensor and effector of hypoxia, HIF1α is vital for the robust transcription regulation of the network genes in hypoxia responses, participating in cellular metabolism, angiogenesis, metastasis, stemness and immune escape [45]. In recent years, vascular normalization therapies have been aimed in part at alleviating hypoxia and inhibition of HIF1α to prevent cancer progression [46]. Many studies have shown that blocking HIF1α enhances the efficacy of chemo-, radio- and immuno-therapies and therefore becomes a potential therapeutic strategy for cancer [47].

Loss of TET1 has been associated with hypermethylation of tumor suppressor genes, thereby leading to their inactivation [48–50]. However, in addition to its such tumor suppressor functions, emerging evidence shows that TET1 can also induce the expression of a series of oncogenes dependent or independent of its dioxygenase activity [18–20], suggesting the existence of its non-enzymatic functions. In the present study, we found that low TET1 expression was strongly associated with better patient survival in different cancers, including thyroid cancer, consistent with in vivo results that *Tet1* knockout in transgenic mouse model of thyroid cancer suppressed tumor progression and prolonged mice survival. However, in vitro experiments under normoxia demonstrated that TET1 overexpression suppressed the proliferation, colony formation and migration of thyroid cancer cells, while TET1 knockout produced opposite effects. These observations suggest that the cancer-related functions of TET1 are situational.

Considering that hypoxia is a common characteristic of solid tumors, we speculated that hypoxia might contribute to the diverse functions of TET1 observed between in vitro and in vivo studies. Indeed, this study demonstrated that hypoxia switched on oncogenic functions of TET1. Moreover, our data indicated that HIF1α was a key determinant for hypoxia-mediated oncogenic functions of TET1. HIF1α is usually degraded via the PHD/VHL pathway under normoxia [51], thereby nulling the promoting effect of TET1 on HIF1α, which explains why TET1 is not oncogenic in normoxia. We also demonstrated that TET1 did not affect *HIF1A* transcription but protected HIF1α from proteasomal degradation under hypoxia, explaining the oncogenic function of TET1 in hypoxia. Interestingly, we found that TET1 increased rather than decreased polyubiquitination of HIF1α, seemingly conflicting with its stability. In fact, there is evidence showing that different composition of ubiquitin chains lead to different outcomes of their substrates [32]. Although the present study showed that TET1 elevated polyubiquitination of HIF1α, K48-linked ubiquitination as a protein degradation signal was suppressed by TET1. Thus, TET1 stabilizes HIF1α under hypoxia by suppressing its K48-linked ubiquitination and degradation.

The present study further demonstrated that CK2 was required for TET1-mediated HIF1α stabilization. CK2 is a ubiquitously expressed and highly conserved protein serine/threonine kinase composed of two α catalytic subunits and two β regulatory subunits [41]. This study demonstrated that TET1 upregulated CK2β expression in a HIF1α-dependent manner and identified *CK2B* as a downstream target of HIF1α, which was consistent with a previous study showing hypoxia-induced CK2β expression [52]. We also found that TET1 overexpression upregulated the protein expression of CK2α under hypoxia at a post-transcriptional level. Previous studies indicated that CK2β was apparently linked to CK2α stability [41]. We speculated that TET1-mediated CK2α upregulation under hypoxia might be attributed to the apparent linkage of CK2β to CK2α stability. In addition, hypoxia has been demonstrated to induce the re-localization of CK2β at the plasma membrane and subsequently maximize CK2α activity [52], where CK2 alters the conformation of PTEN by phosphorylating its C-terminal tail. As a result, the protein stability of PTEN was increased, but its lipid phosphatase activity was decreased [37, 38], thereby leading to increased phosphorylation of AKT at T308 and activation of the AKT signaling. Also, CK2 directly phosphorylates AKT at S129 to fully fuel its activation [39] and fully activated PI3K/AKT pathway prevents the FBW7-mediated K48-linked ubiquitination and degradation of HIF1α by suppressing the GSK3β activity [53]. In line with these observations, our data showed that TET1 phosphorylated and inactivated PTEN by upregulating CK2, thereby stabilizing HIF1α by activating the AKT pathway.

CK2 is highly expressed in various cancers and exerts oncogenic functions by activating several major signaling pathways, such as MAPK/ERK, PI3K/AKT, IKK/NFκB and Wnt/β-catenin pathways [39, 54, 55]. Thus, CK2 is becoming an increasingly attractive therapeutic target in human cancers. In fact, CK2 inhibitor CX-4945 has been approved by the Food and Drug Administration (FDA) of USA for the treatment of cholangiocarcinoma and medulloblastoma and is tested in clinical trials for different cancers [56]. Our present study demonstrated that CX-4945 effectively attenuated the tumor-promoting effects of TET1 in cancer cells under hypoxia, providing a molecular explanation for the anti-cancer effect of CK2 inhibitors and further supporting their therapeutic potential in solid cancers, particularly those with high TET1 expression.

As a member of the DNA dioxygenase family, TET1 is often thought to regulate gene expression via DNA demethylation [57]. The present study showed that TET1 promoted *CK2B* transcription under hypoxia but did not change 5-hmC levels at its promoter. Previous studies have demonstrated that TET1 is enzymatically suppressed under hypoxia [8, 25]. In addition to its enzymatic activity, TET1 also serves as a coactivator/corepressor of transcription factors [58]. There is considerable evidence indicating that p300 is required for the transcriptional activity of HIF1α [59]. The present study demonstrated that TET1 knockout not only impaired the interaction between p300 and HIF1α under hypoxia, but also reduced the enrichment of p300 to the *CK2B* promoter. Our data, taken together, indicate that TET1 functions as a coactivator of HIF1α to mediate its interaction with p300 and consequent promotion of *CK2B* transcription under hypoxia.

In summary, this study demonstrates that TET1 plays distinct roles under in vitro and in vivo conditions in cancer, such as thyroid cancer, which is due to the different oxygen environments; hypoxia switches on its oncogenic functions through HIF1α. In this process, TET1 acts as a coactivator of HIF1α to promote *CK2B* transcription under hypoxia by mediating the interaction between p300 and HIF1α. CK2, at increased expression, activates the AKT pathway to promote oncogenesis. The activated AKT pathway, through suppressing GSK3β, in turn prevents the FBW7γ-mediated K48-linked ubiquitination and degradation of HIF1α, thereby forming a self-enhancement feedback loop. Our work, using thyroid cancer as a model, provides evidence for the oncogenic function switch of TET1 under hypoxia and identifies an underlying mechanism independent of its classical enzymatic activity. This explains the correlation between high TET1 expression and poor clinical outcomes of cancer patients. The oxygen state-governed distinct functions of TET1 demonstrate the importance of tumor microenvironment in determining the roles of key molecules in cancer and provide a rationale for situation-based designing of therapeutic strategies. This study provides implications for novel therapeutic targeting strategies for cancer.

## Materials and methods

### Cell lines and cell culture

Human thyroid cancer cell lines 8505C and K1 were kindly provided by Dr. Haixia Guan (Guangdong Provincial People’s Hospital, Guangzhou, China). Human thyroid cancer cell line C643 was kindly provided by Dr. Lei Ye (Ruijin Hospital, Shanghai, China). The STR DNA profiling of the cell lines was shown in Supplementary Table 2. Cells were cultured in RPMI 1640 (Gibco) supplemented with fetal bovine serum (FBS, 10%; Biological Industries), non-essential amino acids (NEAA) (Gibco), sodium pyruvate (Gibco) and Penicillin-Streptomycin (100 U/l; Gibco). Cell lines were routinely checked for mycoplasma contamination by PCR methods.

### Transgenic mice

*LSL-Braf*^*V600E*^ mice were kindly provide by Dr. Martin McMahon (The University of California, San Francisco). *TPO-Cre* mice were kindly provided by Dr. Shioko Kimura (National Cancer Institute). *LSL-Tet1* mice were purchased from Shanghai Biomodel Organism Science & Technology Development Co., Ltd. A mouse model of thyroid cancer was established according to a previous protocol [27]. *LSL-Braf*^*V600E*^ and *LSL-Tet1* mice were of a B6 genetic background, *TPO-Cre* mice were FVB/NCr. All mice were housed in a certified animal facility with a 12-h light/dark cycle in a temperature-controlled room (25 ± 1 °C) with free access to water and food, in accordance with Swiss guidelines.

### Human materials

With the institutional review board approval and patient consent, seventeen pairs of primary thyroid cancers and their matched noncancerous tissues (control subjects) were collected from the Surgery Section of the First Affiliated Hospital of Xi’an Jiaotong University. RNA and genomic DNA were extracted and prepared from the tissues for TET1 expression and 5-hmC dot-blot analysis. Ten paraffin-embedded thyroid cancers and their matched noncancerous tissues were subjected to IHC staining of TET1. Patients did not receive any therapies before surgery and all tissues were histologically examined by two experienced pathologists at the Department of Pathology of this hospital according to World Health Organization (WHO) criteria.

### Human data sets

The gene expression and survival analysis of cancer patients from The Cancer Genome Atlas (TCGA) were online analyzed at http://ualcan.path.uab.edu/ and http://gepia.cancer-pku.cn/. For Kaplan–Meier analysis of patient’s survival, the data of *TET1* expression were clustered into groups by median values and the overall survival was investigated. The significance for these plots was determined using the Log rank test.

### Transfection of plasmid and short interfering RNAs (siRNA)

The plasmids used for ectopic expression of TET1 and HIF1α were established by inserting the full-length cDNA into pRetroX-tight and pCDNA3.1 vectors, respectively. HRE luciferase reporter plasmid was established based on pGL3-promotor vector (Promega) as described previously [60]. *CK2B* luciferase reporter plasmid was established by inserting its sequence from +14 to −1525 into pGL3-basic (Promega). pCI-K48-Ub-6×His plasmid was kindly provided by Prof. Wuhan Xiao (Chinese Academy of Sciences, Hubei, China), and used for in vitro protein ubiquitination assay. siRNA targeting HIF1α and control siRNA were purchased from Santa Cruz. Cells were transfected with the above plasmids and siRNAs following a standard protocol with Lipofectamine 3000 (Thermo Fisher Scientific, USA).

### Generation of TET1 knockout cell line with CRISPR-Cas9 and single-cell colony

Lentivirus expressing Cas9 and sgRNAs targeting TET1 obtained from Genechem (Shanghai, China) were used to disrupt the *TET1* gene’s activity. The sgRNA sequences were presented in Supplementary Table 3. Their off-target effect was evaluated according to previously reported method and shown in Supplementary Table 3 [61, 62]. Virus transduction was performed with MOI of 10 and polybrene was used during the transduction. To generate cell lines totally delete *TET1*, we performed dilution plating to isolate and expanse single-cell colonies after virus transduction. Sanger sequencing of cut-site PCR products and western blot was performed to identify the colonies successfully knock out *TET1*, which were used in the further studies.

### Western blot analysis

RIPA buffer was used to lyse cell pellets immediately harvested from normoxia or hypoxia cultured cells. Denatured protein lysates were separated by SDS gel electrophoresis and transferred to PVDF membranes (Roche Diagnostics) or NC membranes (Bio-rad). After blocking in BSA solution, the membranes were incubated overnight with the primary antibodies listed in Supplementary Table 4. This was followed by incubation with their respective horseradish peroxidase-conjugated secondary antibodies from ZSGB-BIO, and immunoblotting signals were visualized using the ECL detection kit WBKLS0500 (Millipore).

### Genomic DNA preparation and dot blot assay

Phenol-chloroform extracted genomic DNA from frozen tissues and cancer cells were subjected to dot blot assays to compare 5-hmC levels. The amounts of 250 ng, 100 ng and 50 ng genomic DNA were arranged for dot blot assay of tissues, while 1 μg genomic DNA was subjected to dot blot assay of cell lines. All DNA samples were diluted in 2×SSC buffer and underwent thermal denaturation, followed by ice chilling immediately. DNA samples were spotted onto positive charged nylon membrane (Roche Diagnostics, Mannheim, Germany), fixed by UV irradiation (HL-2000 HybriLinker Hybridization Oven; CA, U.S.A), washed and blocked with 5% casein blocking buffer, and incubated with anti-5-hmC antibody (Active Motif) at 4 °C overnight, followed by incubation with species-specific HRP-conjugated secondary antibody (ZSGB-BIO). Dot signal was then visualized with ECL detection kit WBKLS0500 (Millipore). To ensure equal spotting of total DNA on the membrane, the same blot was stained with 0.02% methylene blue in 0.3 M sodium acetate (pH 5.2).

### Cell proliferation and migration assays

The RTCA instrument iCELLigence was used to assess the adherence property of cancer cells during proliferation. Cells (15,000 cells per well) were seeded in E-16 plates after background measurements and the system was set in incubator for culturing in normoxia or hypoxia. RTCA Software Package 1.2 was used to read the impedance signals produced by gold plated sensor electrodes in the bottom of the plates every 10 min until the end of the experiment. For migration analysis, cells were seeded in upper chamber of CIM-plates with serum free medium. Serum abundant medium was prepared for the lower chamber. The chambers were then mounted together and arranged in incubator to allow cells migrate through the bottom membrane of the upper chamber where gold sensor electrodes were placed. After equilibration and background reading, the impedance signals were recorded 25 scans at 5 min intervals and then followed by scans at every 10 min intervals until the end of the experiment.

### Colony formation assay

Cells (500/well) were seeded in 6-well plates. The medium was changed every 3 days. After 14 days culturing, surviving colonies (≥50 cells per colony) were fixed with methanol and stained with 0.5% crystal violet, pictured and counted. Each experiment was carried out in triplicate.

### Animal studies

All animal experiments were performed with the approval by the Animal Ethics Committee of Xi’an Jiaotong University. Hybrid pups of transgenic mice were subjected to tail tip DNA extraction and genotype identification at 3-weeks age (data not shown). In a pre-experiment, we found that *Braf*^*V600E*^ mutated mice developed thyroid cancer at ~6-weeks age. The early onset of cancer was due to the expression of *TPO-Cre* in embryo day 14.5 which initiates *Braf*^*V600E*^ mutation at very early period of mouse life. As a result, all the thyroid follicular epithelial cells undergo transformation, which was in line with a previous study [27]. During breeding, the time of mouse natural death was recorded and body weight was measured weekly until 100 days of age.

Some of the mice were subjected to iodine uptake examination. Preparation, dilution, injection, and examination of ^131^I-NaI were performed in the Department of Nuclear Medicine of the First Affiliated Hospital of Xi’an Jiaotong University. Approximately 250 μCi of ^131^I-NaI (Institute of Nuclear Physics and Chemistry, China Academy of Engineering Physics) in 200 μl isotonic saline was administered to each mouse via intraperitoneal injection. Mice were sacrificed 6 h after injection and operated to separate thyroid which was put in an Eppendorf tube and applied to radioisotope activity measurements by CRC^®^-15R Dose Calibrator (Miron Technologies). Iodine uptake rate was calculated as follows: % injected Dose/g = (iodine radiation/injected radiation × 100%)/thyroid weight. After 100 days housing, mice with different genotypes were sacrificed and thyroid glands were photoed and weighed. Harvested thyroid tissues were used to extract genomic DNA and protein, and paraffin-embedded tissues was stored until use. Sample size estimation was performed using software of Power and Sample Size Calculation (Vanderbilt University).

### Immunohistochemistry

Isolated tissues were fixed in 10% neutral buffered formalin, embedded in paraffin, sectioned (4 µm) and stained immunohistochemically for different antigens. Slides were deparaffinized, rehydrated and quenched in 0.6% hydrogen peroxide/methanol for 15 min, and boiled for 20 min in 10 mM sodium citrate (pH 6.0) for antigen retrieval. Sections were blocked for 1 h, incubated with primary antibodies overnight at 4 °C, incubated with secondary antibodies next day, followed by developing with 3,3′-diaminobenzidine (ZSGB-BIO). Primary antibodies used are listed in Supplementary Table 4. Slides were counterstained with hematoxylin, dehydrated and covered with glass. All images were captured with a microscope slide scanner (Leica MP, SCN400).

HIF1α and Ki67 positive cells were quantified by using HistoQuest image analysis software (TissueGnostics). Data were shown in percent positivity. For evaluation of phosphorylated Akt, Gsk3β, and Pten, along with Ck2β expression, we qualitatively scored the images. Score ranks in a range, including “negative” (−), “weak” (+), “moderate” (++), and “strong” (+++). The results were presented as the proportion of each score for different groups.

### CHX chase assay

Cells were treated with 200 μg/ml CHX (MP Biomedicals) to stop de novo protein synthesis. At the indicated time points, cell lysates were harvested and then subjected to immunoblotting.

### Dual-luciferase reporter system

We first constructed pGL3-promoter-HRE-Luc and pGL3-Basic-CK2β-Luc plasmids. Cancer cells were then co-transfected with different Luc plasmids and pRL-TK (Promega) together with HIF1α siRNA or overexpression plasmids if necessary. Cell lysates were collected 48 h post-transfection. Luciferase activity was analyzed using the dual-luciferase reporter system (Promega) and visualized by EnSpire Multimode Plate Reader (PerkinElmer). Data were shown as relative luciferase activity (Firefly luciferase activity/Renilla luciferase activity). Each experiment was run in triplicate.

### Co-immunoprecipitation (Co-IP)

Cell lysates were incubated with antibodies against target proteins or IgG at 4 °C for 3 h, followed by incubation with protein A/G-agarose beads (Santa Cruz Biotechnology) at 4 °C overnight. Immunoprecipitates were washed with RIPA buffer, and then subjected to western blot analysis.

### In vitro protein ubiquitination assay

Cells were subjected to the indicated treatments, including TET1 modulation, proteasome inhibition by MG132, GSK3β inhibition by SB216763, and ectopic expression of His-tagged K48-only ubiquitin. Cell lysates were harvested and incubated with 1 μl anti-HIF1α antibody and Protein A/G PLUS-Agarose (Santa Cruz Biotechnology, Inc.). The finally eluted proteins were immunoblotted with an anti-ubiquitin antibody (Abcam) to detect intrinsic ubiquitin, or with anti-His tag antibody (Cell Signaling Technology) for ectopic K48-only ubiquitin.

### Phosphatidylinositol-3,4,5-trisphosphate (PIP3) phosphatase assay

The PIP3 phosphatase assay was performed to detect cellular PTEN activity using colorimetry assay kit (GMS50064.1, Genmed, Shanghai) following the manufacturer’s protocol. Briefly, 5 × 10^7^ cells were washed and lysates harvested with supplied lysis buffer (Reagent B). The samples were then simultaneously incubated with reagents rendering extrinsic PIP3 (Reagent E) or not (Reagent C) at 37 °C for 10 min. The reactions were stopped by Reagent F buffer. Reagent G buffer for color developing was added and incubated in the room temperature in dark for 15 min. Both intrinsic and rendered PIP3 were dephosphorylated by PTEN to release free phosphates, which reacted with malachite green dye and was measured by spectrophotometer at 660 nm. The difference between the phosphates produced by the reagents E and C represented idle PTEN activities in the cells. Gradient concentration of standard samples was used to establish calibration and standard curve. PTEN activities were finally visualized as the phosphates produced per minute per milligram of sample (nmol Pi/min/mg).

### RNA isolation and qRT-PCR assay

Total RNA was isolated from tissues and cell lines using TRIZOL reagent (Takara, Inc.) and was converted to cDNA using PrimeScript RT reagent Kit (Takara, Inc.) according to the manufacturer’s protocol. qRT-PCR was carried out on a CFX96 Thermal Cycler real-time PCR system (Bio-Rad Laboratories, Hercules, CA) using SYBR Premix Ex Taq (Takara, Inc.). *GAPDH* was used as a reference gene to normalize target gene mRNA levels. Three triplicates were done for each sample. The sequences of primers are listed in Supplementary Table 5.

### ChIP-qPCR assay

The ChIP-qPCR assay was performed to evaluate the enrichment of HIF1α, TET1 and p300 on *CK2B* promoter using the SimpleChIP^®^ Enzymatic Chromatin IP Kit (Cell Signaling Technology) following the manufacture’s protocol. Briefly, the harvested cells (~4 × 10^6^ cells) were cross-linked using formaldehyde (final concentration 1% vol/vol) for 10 min at room temperature, followed by quenching with glycine (final concentration 0.125 M) for 5 min at room temperature. The cells were collected in PBS buffer and subjected to nuclei isolation. Prepared nuclei were digested with micrococcal nuclease for 10 min and the lysates were sonicated with VCX-130PB (Sonics & Materials, Inc., Newtown, CT, USA) to fragment the chromatin to an average size of 300–500 bp. About 2% of total chromatin from each lysate was maintained as input control and the remaining chromatin was incubated overnight with 1–10 μg of indicated antibodies respectively in ChIP Buffer. Non-specific IgG was used as control. Immunoprecipitated protein DNA complex was then isolated with ChIP-Grade Protein G Magnetic Beads at 4 °C for 2 h. Chromatin was eluted in ChIP Elution Buffer, and the proteins were removed with the addition of 200 mM NaCl and proteinase K (200 μg/ml) at 65 °C for 2 h. DNA of input and immunoprecipitated samples were purified and used as templates for further analysis. Primer sequences for ChIP-qPCR assay were listed in Supplementary Table 5. The PCR products were then subjected to agarose gel electrophoresis. Each test was run in triplicate.

### hMeDIP-qPCR assay

Sonication fragmented DNA were prepared in IP buffer. After thermal denaturation and immediately chilling, 10% sample was saved as the input control. DNA fragments were then incubated with anti-5-hmC antibody at 4 °C overnight, followed by protein A/G agarose beads precipitation. After washing, DNA components were eluted by SDS containing alkaline solution from the precipitates. DNA was purified by phenol chloroform extraction and stored for qPCR analysis. The sequences of primers are presented in Supplementary Table 5.

### Statistical analysis

Data were compared using the Student’s tests (SPSS Statistics for Windows v16.0, Chicago, IL). All values were expressed as the mean ± standard deviation (SD). Log rank tests (SPSS Statistics for Windows v16.0, Chicago, IL) were used to investigate the Kaplan–Meier curves (SPSS Statistics for Windows v16.0, Chicago, IL). A *p* value of <0.05 was considered to be statistically significant. Unless indicated otherwise, the data shown in the figures are representative examples. Significance indication: *p < 0.05; **p < 0.01; ***p < 0.001; ns, non-significant.

## Supplementary information

Supplementary Information

## Data Availability

All relevant data are available from the authors upon request.
