# Supplementary material for: Hypoxia switches TET1 from being tumor-suppressive to oncogenic
Source: Oncogene. 2023 Apr 5;42(20):1634–48. doi: 10.1038/s41388-023-02659-w (PMC10181935; doi:10.1038/s41388-023-02659-w)
Supplement: Supplementary file 1 — Supplementary Information [file 41388_2023_2659_MOESM1_ESM.docx]

**Hypoxia switches TET1 from being tumor-suppressive to oncogenic**

Qi Yang^1,2,3^, Hui Dang^2^, Jiaxin Liu^1^, Xingye Wang^1,4^, Jingyuan Wang^1,5^, Xinhui Lan^2^, Meiju Ji^1,6,*^, Mingzhao Xing^7,*^ and Peng Hou^1,2,*^

^1^ Key Laboratory for Tumor Precision Medicine of Shaanxi Province, The First Affiliated Hospital of Xi’an Jiaotong University, Xi’an 710061, P.R. China

^2^ Department of Endocrinology, The First Affiliated Hospital of Xi’an Jiaotong University, Xi’an 710061, P.R. China

^3^ Department of Otorhinolaryngology-Head and Neck Surgery, The First Affiliated Hospital of Xi’an Jiaotong University, Xi’an 710061, P.R. China

^4^ Department of Structural Heart Disease, The First Affiliated Hospital of Xi'an Jiaotong University, Xi'an 710061, P.R. China

^5^ Department of Clinical Laboratory, The First Affiliated Hospital of Xi'an Jiaotong University, Xi'an 710061, P.R. China

^6^ Center for Translational Medicine, The First Affiliated Hospital of Xi'an Jiaotong University, Xi'an 710061, P.R. China

^7^ School of Medicine, Southern University of Science and Technology, Shenzhen, Guangdong 518055, P.R. China

*** Corresponding authors:**

E-mail (Mingzhao Xing): [xingmz@sustech.edu.cn](mailto:xingmz@sustech.edu.cn)

E-mail (Peng Hou): [phou@xjtu.edu.cn](mailto:phou@xjtu.edu.cn)

E-mail (Meiju Ji): mjji0409@163.com

**Table of Contents**

Supplementary Fig. 1…………………………………………………………………….S3

Supplementary Fig. 2…………………………………………………………………….S4

Supplementary Fig. 3…………………………………………………………………….S5

Supplementary Fig. 4…………………………………………………………………….S7

Supplementary Fig. 5…………………………………………………………………….S9

Supplementary Fig. 6………………………………………………………………...…S10

Supplementary Table 1 Single cell colonies used in this study……………………....S12

Supplementary Table 2 The STR DNA profiling of cell lines used in this study…….S12

Supplementary Table 3 sgRNA sequences used in this study………………………...S13

Supplementary Table 4 Antibodies used in this study………………………………..S14

Supplementary Table 5 Primers used in this study…………………………………...S15


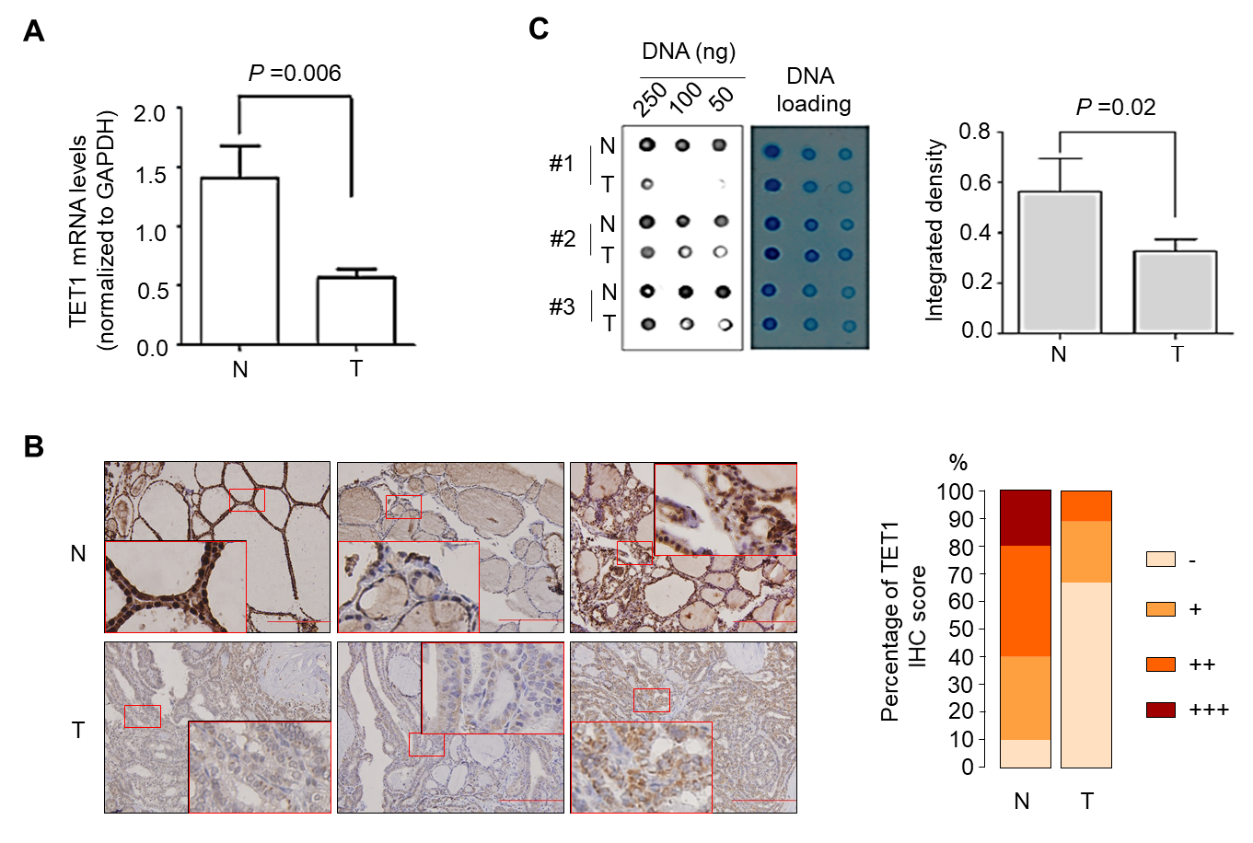
**Supplementary Fig. 1** Decreased expression of TET1 in PTCs compared to their paired noncancerous tissues. Seventeen pairs of primary PTCs (T) and their distant noncancerous thyroid tissues (N) were collected. **A** Freshly extracted RNA samples were prepared for *TET1* mRNA analysis. *GAPDH* was used as a reference gene. Graph shows mean ± SD; unpaired Student’s test. **B** Paraffin embedded PTCs and normal tissues were sectioned and subjected to the IHC staining of TET1. Sections were rank scored [“negative” (−), “weak” (+), “moderate” (++), and “strong” (+++)]. Graph shows the proportion of each score for different groups; Scale bare, 50 μm. **C** Dot blot analysis of 5-hmC was used to evaluate genomic 5-hmC levels of PTCs and normal tissues. Loading amounts were visualized by the methylene blue staining. Integrated density of 5-hmC dots was calibrated with a doubling diluted standard. Graph shows mean ± SD; unpaired Student’s test.

**
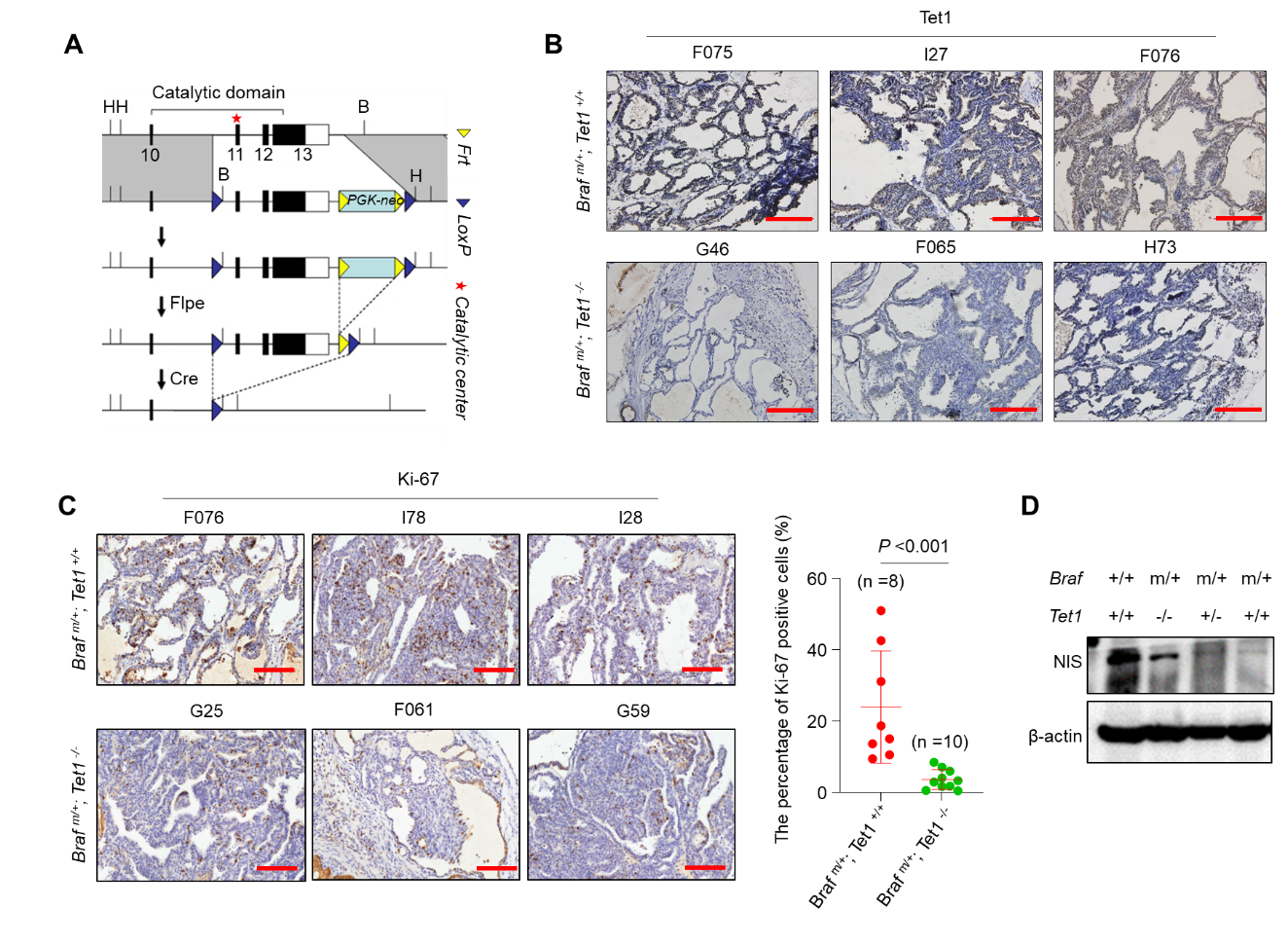
Supplementary Fig. 2** Tet1 knockout inhibits cellular mitosis of murine PTC. **A** The schematic diagram shows *Tet1* gene manipulation in *LSL-Tet1* mouse. **B** IHC staining shows Tet1 expression status in thyroid tumor tissues of *Braf ^m/+^; Tet1^+/+^* and *Braf ^m/+^; Tet1^-/-^* mice. Scale bare, 50 μm. **C** IHC staining of Ki-67 in murine PTC tissues shows cellular mitosis rate. The positivity of Ki-67 was shown in the right panel. Graph shows mean ± SD; unpaired Student’s test; Scale bare, 50 μm. **D** Western blotting shows the expression of the sodium iodine transporter (NIS) in mice with the indicated genotypes.

**
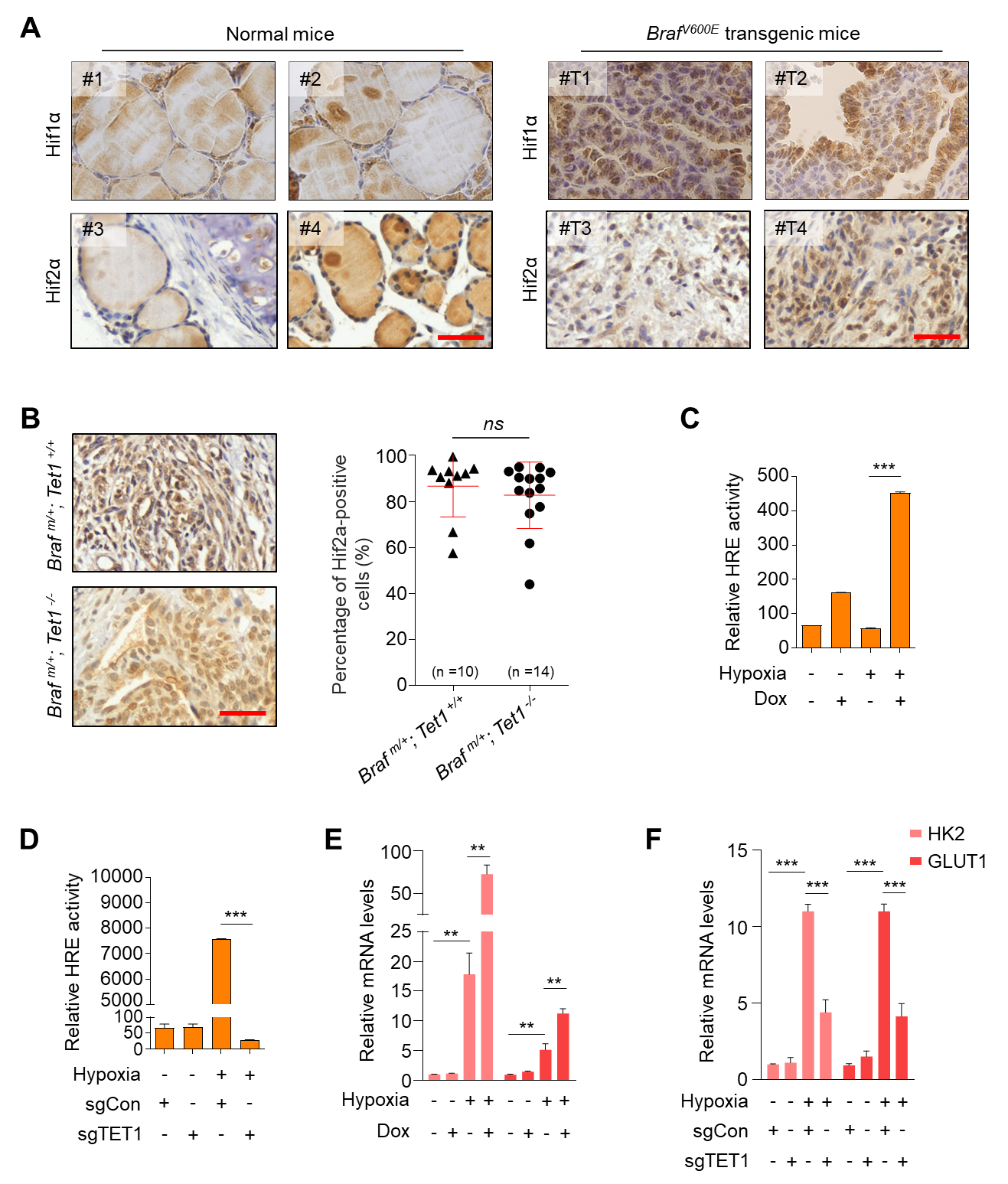
Supplementary Fig. 3 A** The IHC staining of Hif1α and Hif2α in normal murine thyroid and mouse thyroid cancer tissues. Hif1α and Hif2α in the nucleus is shown in brown, if present. Scale bare, 50 μm. **B** IHC staining of Hif2α and its quantification in thyroid cancer tissues of the indicated mice. HRE luciferase activity in 8505C (**C**) and C643 (**D**) cells with the indicated treatments. Graph shows relative luciferase activity (Firefly/*Renilla*) as mean ± SD; unpaired Student’s test; ***, *P* < 0.001. qRT-PCR assay was performed to analyze transcription levels of HIF1α target genes *HK2* and *GLUT1* in 8505C (**E**) and C643 (**F**) cells with the indicated treatments. Graph shows mean ± SD; *GAPDH* was used as a reference gene. Hypoxia, 1% oxygen incubation for 24 h; Dox-, negative control; Dox+, cells inducibly expressing TET1; sgCon, control cells; sgTET1, C643 cells knocking out TET1; unpaired Student’s test; *ns*, no significant; ***P* < 0.01, ****P* < 0.001.

**
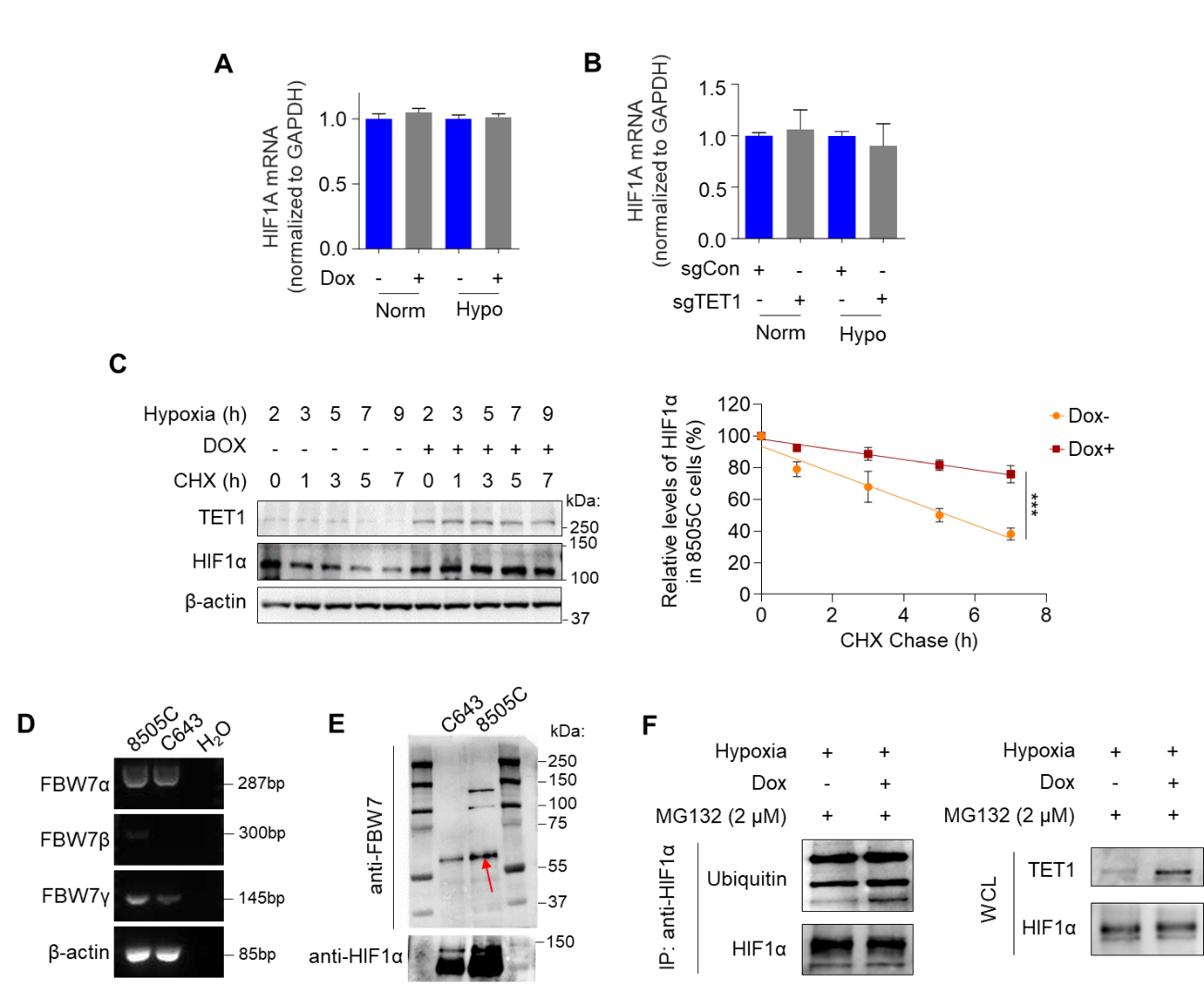
Supplementary Fig. 4** mRNA expression of *HIF1A* in 8505C (**A**) and C643 (**B**) cells with the indicated treatments. *GAPDH* was used as a reference gene. Graph shows Dmean ± SD; unpaired Student’s test shows no significant differences. **C** Western blot analysis of TET1 and HIF1α in C643 cells with the indicated treatments. The band intensity of HIF1α was normalized to that of β-actin, and subsequently normalized to that of cells treated without CHX. Data are shown as the mean ± SD. **D** PCR products specifically amplifying mRNA sequences of FBW7α, β and γ as well as β-actin were subjected to agarose gel electrophoresis. **E** The IP products of anti-HIF1α were detected by western blotting using FBW7 antibody. The red arrow indicates the specific band of FBW7γ.  **F** *In vitro* protein ubiquitination analysis of HIF1α in 8505C cells with the indicated treatments. Left panel shows the precipitated HIF1α and ubiquitin on HIF1α. Right panel shows the whole cell lysate (WCL) as reference. Hypoxia, 1% oxygen incubation for 24 h; Dox-, negative control; Dox+, cells inducibly expressing TET1; sgCon, control cells; sgTET1, C643 cells knocking out TET1.

**
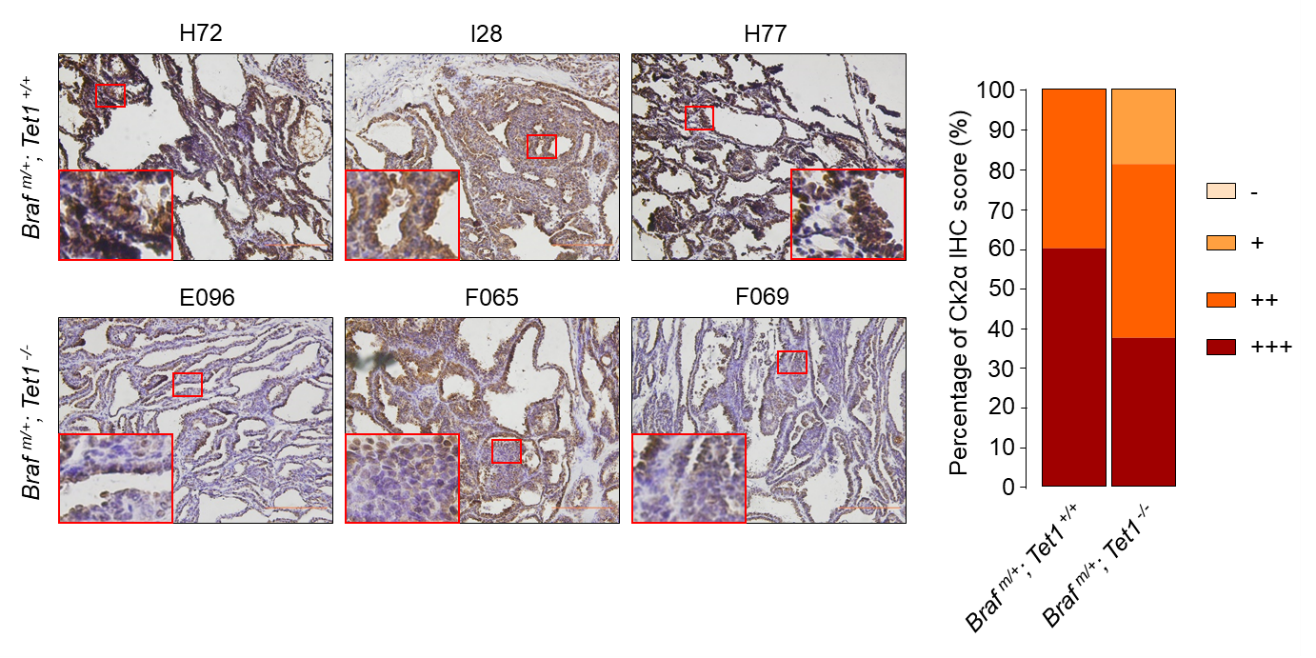
Supplementary Fig. 5** The IHC staining of Ck2α (left panel) and its quantification (lower panel) in mice with the indicated genotypes. Rank scores were estimated [i.e., “negative” (−), “weak” (+), “moderate” (++), and “strong” (+++)]. Graph shows the proportion of each score for different groups; Scale bare, 50 μm. Braf^m/+^, *Braf* heterozygous mutation; Tet1^+/+^, *Tet1* wild-type; Tet1^-/-^, *Tet1* homozygous deletion.

**
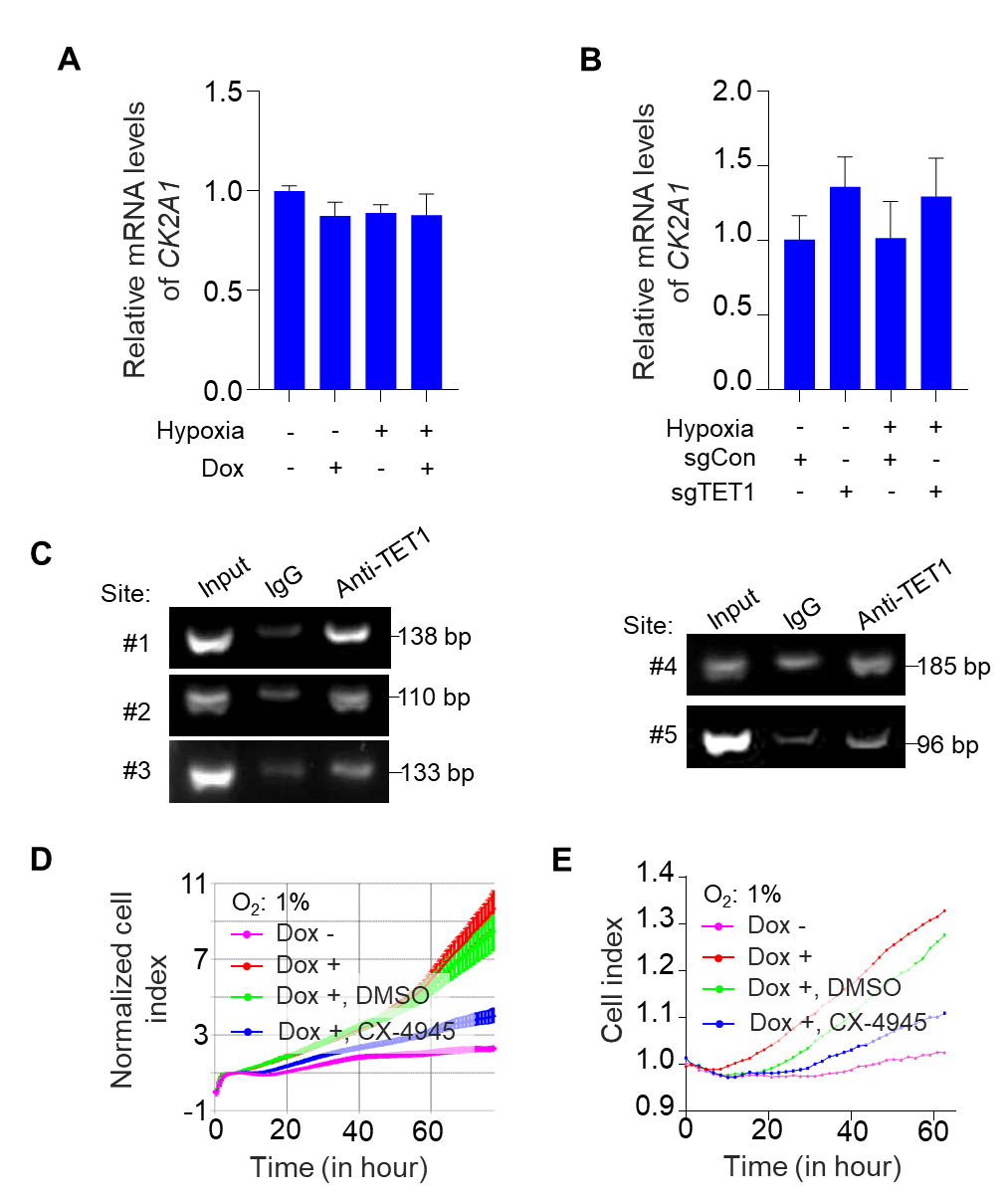
Supplementary Fig. 6** mRNA expression of *CK2A1* in 8505C (**A**) and C643 (**B**) cells with the indicated treatments. *GAPDH* was used as a reference gene. Graph shows mean ± SD; unpaired Student’s test. Dox-, negative control; Dox+, cells inducibly expressing TET1; sgCon, control cells; sgTET1, C643 cells knocking out TET1. **C** *CK2B* promoter sequences were amplified from TET1-associated chromatins of C643 cells cultured under hypoxia. The products were then subjected to agarose gel electrophoresis. In 8505C cells, TET1 expression were induced by Dox, and treated with CK2 inhibitor (CX-4945, 4 μM) or DMSO. The proliferation (**D**) and migration (**E**) of the above cells were recorded by RTCA under hypoxia. Dox-, negative control; Dox+, cells inducibly expressing TET1.

**Supplementary Table 1** Single cell colonies used in this study

| **Clone ID** | **Allele 1** | **Allele 2** | **Generation Method** |
| --- | --- | --- | --- |
| sgTET1#1 | 7bp deletion from 68,645,333 | 24bp deletion from 68,645,332 | CRISPR-CAS9 knockout using #1 sgTET1 |
| sgTET1#2 | 12bp deletion from 68,572,357 | 12bp deletion from 68,572,357 | CRISPR-CAS9 knockout using #2 sgTET1 |

**Supplementary** **Table 2** The STR DNA profiling of cell lines used in this study

| **Cell lines** | **Amelo** | **D13S317** | **D18S51** | **D21S11** | **D3S1358** | **D5S818** | **D7S820** | **D8S1179** | **FGA** | **vWA** |
| --- | --- | --- | --- | --- | --- | --- | --- | --- | --- | --- |
| K1 | X/Y | 11/14 | 18/18 | 30/31.2 | 18/18 | 10/11 | 11/11 | 15/15 | 21/24 | 17/18 |
| C643 | X/Y | 8/10 | 14/18 | 28/28 | 15/15 | 11/12 | 9/12 | 11/13 | 18/21 | 15/17 |
| 8505C | X/X | 13/13 | 16/16 | 28/32.2 | 16/17 | 10/11 | 10/10 | 10/13 | 23/23 | 17/19 |

**Supplementary** **Table 3** sgRNA sequences used in this study

| **Oligonucleotides (5’-3’)** | **Off-target score** |
| --- | --- |
| #1 sgRNA targeting TET1: TGAACGGGAGATCCATCTTT | 77.1 |
| #2 sgRNA targeting TET1: TGTCTCGATCCCGCCATGCA | 90.9 |

**Supplementary** **Table 4** Antibodies used in this study

| **Antigen** | **Source** | **Identifier** |
| --- | --- | --- |
| TET1 | GeneTex | GTX124207 |
| β-actin | Abcam | ab28277 |
| 5-hmC | Active Motif | 39769 |
| HIF1α | Novus Biologicals | NB100-134 |
| HIF2α | Santa Cruz | SC-46691 |
| p-AKT^T308^ | Bioworld | AP0056 |
| p-AKT^S473^ | Bioworld | BS4006 |
| t-AKT | Cell Signaling Technologies | #4691 |
| p-GSK3β S9 | Santa Cruz | SC-373800 |
| GSK3α/β | Santa Cruz | SC-7291 |
| GSK3β | Cell Signaling Technologies | #9336 |
| p-β-catenin^S33/37/T41^ | Cell Signaling Technologies | #9561 |
| β-catenin | Santa Cruz | sc-133240 |
| FBW7 | Santa Cruz | sc-293423 |
| VHL | Santa Cruz | sc-135657 |
| His tag | Cell Signaling Technologies | #2365 |
| PTEN | Santa Cruz | sc-7974 |
| p-PTEN^S370^ | Abcam | ab195056 |
| CK2α | Santa Cruz | sc-373894 |
| CK2β | Abcam | CK2β |
| p300 | Abcam | ab14984 |
| Ki67 | Santa Cruz | sc-23900 |
| Ubiquitin | Abcam | ab7780 |

**Supplementary** **Table 5** Primers used in this study

| **Targets** | | **Oligonucleotides (5’- 3’)** | **Application** |
| --- | --- | --- | --- |
| TET1 | Forward | GCCAACCTTAGGGAGTAACAC | RT-qPCR |
|  | Reverse | TTGCGTCATTCTTCAGTGGA | RT-qPCR |
| HK2 | Forward | AGCCCTTTCTCCATCTCCTT | RT-qPCR |
|  | Reverse | AACCATGACCAAGTGCAGAA | RT-qPCR |
| GLUT1 | Forward | CTTTGTGGCCTTCTTTGAAGT | RT-qPCR |
|  | Reverse | CCACACAGTTGCTCCACAT | RT-qPCR |
| HIF1α | Forward | GTTCACCTGAGCCTAATAGTCC | RT-qPCR |
|  | Reverse | CCAAGTCTAAATCTGTGTCCTG | RT-qPCR |
| CKA1 | Forward | GGCTTACTGCAAGAGAGGCA | RT-qPCR |
|  | Reverse | GAAGGGGTTGGCACTGAAGA | RT-qPCR |
| CK2B | Forward | TGGAGCCTGATGAAGAACTG | RT-qPCR |
|  | Reverse | GGACAGTAACCAAAGTCTCCT | RT-qPCR |
| GAPDH | Forward | CATTTCCTGGTATGACAACGA | RT-qPCR |
|  | Reverse | GCACAGGGTACTTTATTGATGG | RT-qPCR |
| FBW7α | Forward | GAACCAACCCACTTTCTCGG | RT-qPCR |
|  | Reverse | TTCTACTCCAACAACTTCACCA | RT-qPCR |
| FBW7β | Forward | GGTTCTGCTCCCTAATCTTCC | RT-qPCR |
|  | Reverse | CCAGTGGTACTTGTATATTCTGAG | RT-qPCR |
| FBW7γ | Forward | GCCTCTACCACATCAAACTG | RT-qPCR |
|  | Reverse | CTTTGCATGGTTTCTTTCCC | RT-qPCR |
| CK2B promoter | #1 Forward | GGGATTGGTAGTTCGCTTTCTC | ChIP, hMeDIP |
|  | #1 Reverse | GGGAGTGGAAATTAGGGAGG | ChIP, hMeDIP |
|  | #2 Forward | CCAACAGGCAATAAGGACCC | ChIP, hMeDIP |
|  | #2 Reverse | GATCTCTTTCCGCAGCTCTC | ChIP, hMeDIP |
|  | #3 Forward | GCACTAGGATCTGTCGGTTGG | ChIP, hMeDIP |
|  | #3 Reverse | GGCCTTTCCTAACCCACAGAG | ChIP, hMeDIP |
|  | #4 Forward | ATCAAATAGCCACACGGCAC | ChIP, hMeDIP |
|  | #4 Reverse | CGACAGATCCTAGTGCTCGC | ChIP, hMeDIP |
|  | #5 Forward | GGTCACTCCCTAGAAGATCTC | ChIP, hMeDIP |
|  | #5 Reverse | TCAACTCTTCTGATTAGAATGGCG | ChIP, hMeDIP |
